# Temperate southern Australian coastal waters are characterised by surprisingly high rates of nitrogen fixation and diversity of diazotrophs

**DOI:** 10.7717/peerj.10809

**Published:** 2021-03-01

**Authors:** Lauren F. Messer, Mark V. Brown, Paul D. Van Ruth, Mark Doubell, Justin R. Seymour

**Affiliations:** 1 Climate Change Cluster, University of Technology Sydney, Sydney, New South Wales, Australia; 2 Centre for Microbiome Research, School of Biomedical Sciences, Queensland University of Technology, Brisbane, QLD, Australia; 3 School of Environmental and Life Sciences, University of Newcastle, Callaghan, New South Wales, Australia; 4 Aquatic Sciences, South Australian Research and Development Institute, Adelaide, South Australia, Australia

**Keywords:** Nitrogen fixation, Inverse estuary, Diazotroph dynamics, Microbial ecology, Temperate coastal waters

## Abstract

Biological dinitrogen (N_2_) fixation is one mechanism by which specific microorganisms (diazotrophs) can ameliorate nitrogen (N) limitation. Historically, rates of N_2_ fixation were believed to be limited outside of the low nutrient tropical and subtropical open ocean; however, emerging evidence suggests that N_2_ fixation is also a significant process within temperate coastal waters. Using a combination of amplicon sequencing, targeting the nitrogenase reductase gene (*nifH*), quantitative *nifH* PCR, and ^15^N_2_ stable isotope tracer experiments, we investigated spatial patterns of diazotroph assemblage structure and N_2_ fixation rates within the temperate coastal waters of southern Australia during Austral autumn and summer. Relative to previous studies in open ocean environments, including tropical northern Australia, and tropical and temperate estuaries, our results indicate that high rates of N_2_ fixation (10–64 nmol L^−1^ d^−1^) can occur within the large inverse estuary Spencer Gulf, while comparatively low rates of N_2_ fixation (2 nmol L^−1^ d^−1^) were observed in the adjacent continental shelf waters. Across the dataset, low concentrations of NO_3_/NO_2_ were significantly correlated with the highest N_2_ fixation rates, suggesting that N_2_ fixation could be an important source of new N in the region as dissolved inorganic N concentrations are typically limiting. Overall, the underlying diazotrophic community was dominated by *nifH* sequences from Cluster 1 unicellular cyanobacteria of the UCYN-A clade, as well as non-cyanobacterial diazotrophs related to *Pseudomonas stutzeri*, and Cluster 3 sulfate-reducing deltaproteobacteria. Diazotroph community composition was significantly influenced by salinity and SiO_4_ concentrations, reflecting the transition from UCYN-A-dominated assemblages in the continental shelf waters, to Cluster 3-dominated assemblages in the hypersaline waters of the inverse estuary. Diverse, transitional diazotrophic communities, comprised of a mixture of UCYN-A and putative heterotrophic bacteria, were observed at the mouth and southern edge of Spencer Gulf, where the highest N_2_ fixation rates were observed. In contrast to observations in other environments, no seasonal patterns in N_2_ fixation rates and diazotroph community structure were apparent. Collectively, our findings are consistent with the emerging view that N_2_ fixation within temperate coastal waters is a previously overlooked dynamic and potentially important component of the marine N cycle.

## Introduction

By providing a source of new nitrogen (N), dinitrogen (N_2_) fixation, the microbially mediated conversion of N_2_ gas to ammonia, represents a fundamental process within oligotrophic marine environments (Eugster & Gruber, 2012; Karl et al., 2012). Based on global ocean estimates, the activity of N_2_ fixing microorganisms (termed diazotrophs) contributes approximately 160 Tg of new N to the ocean annually (Wang et al., 2019). However, the majority of empirical observations contributing towards global N_2_ fixation estimates have been derived from tropical and subtropical oceanic gyres (Luo et al., 2012), which have traditionally been deemed the principal ecological niche for marine N_2_ fixation due to the activity of large filamentous and heterocystous cyanobacterial diazotrophs (Breitbarth, Oschlies & LaRoche, 2007; Goebel et al., 2010; Karl et al., 2002). In contrast, temperate coastal habitats have generally been thought to be enriched in dissolved inorganic N derived from terrestrial and upwelled sources (Jickells, 1998), thereby restricting the niche for biological N_2_ fixation.

Temperate coastal waters are some of the most productive regions on Earth, which have historically been believed to be fuelled by the influx of bioavailable nutrients from rivers, groundwater, and through the mixing of offshore waters (Jickells, 1998). Often these hydrodynamic properties result in the development and maintenance of relatively eutrophic conditions, which in combination with typically cool sea surface temperatures, resulted in the supposition that diazotrophic growth and activity, particularly for the large filamentous cyanobacterium *Trichodesmium sp.*, would be limited (Breitbarth, Oschlies & LaRoche, 2007; Howarth et al., 1988; Knapp, 2012). However, largely due to the newly recognised abundance and activity of non-cyanobacterial diazotrophs and unicellular cyanobacteria (UCYN) outside of the traditional oceanic niches of N_2_ fixation, there has been a recent paradigm shift in the potential importance of N_2_ fixation in temperate coastal regions, where annual N_2_ fixation rates have been estimated to exceed 16 Tg N (Tang et al., 2019). Therefore, an enhanced understanding of N_2_ fixation rates and patterns in diazotroph diversity and activity within temperate coastal habitats is required to inform models of marine N availability at regional and global scales.

The widespread application of molecular surveys targeting the gene encoding a subunit of the nitrogenase enzyme complex (*nifH*), have greatly expanded the known diversity and global distribution of numerically important diazotrophs (Cornejo-Castillo et al., 2018; Farnelid et al., 2011; Langlois et al., 2015; Moisander et al., 2010; Zehr et al., 1998; Zehr, Carpenter & Villareal, 2000; Zehr et al., 2003). For example, *nifH* containing UCYN clades, *Candidatus* Atelocyanobacterium thalassa (UCYN-A), UCYN-B, and UCYN-C, and putative heterotrophic, non-cyanobacterial diazotrophs, from the gamma-, delta-, and alphaproteobacteria, have recently been detected throughout the major ocean basins (Díez et al., 2012; Farnelid et al., 2013; Fernández-Méndez et al., 2016; Gradoville et al., 2017; Langlois et al., 2015). Investigations into the ecology of these novel diazotrophs have revealed a range of physiologies and patterns of biogeography. Both free-living (e.g., UCYN-B, and C; Zehr & Turner, 2001; Taniuchi et al., 2012; Stenegren et al., 2018) and symbiotic (e.g., UCYN-A) UCYN groups have been identified, and evidence suggests a diversity of closely related sub-lineages are representative of distinct ecological niches typically associated with “open ocean” (e.g., UCYN-A1) and “coastal” (e.g., UCYN-A2) environments (Cornejo-Castillo et al., 2018; Farnelid et al., 2016; Thompson et al., 2014; Farnelid et al., 2016).

The isolation of non-cyanobacterial diazotrophs from specific habitats, such as the Peruvian oxygen minimum zone (Martínez-Perez et al., 2018), and estuarine waters of the Baltic Sea (Bentzon-Tilia et al., 2014; Farnelid et al., 2014), imply relatively specialised niches for these organisms. However, genomic analysis of isolates, and metagenome-assembled genomes from the alphaproteobacteria and Planctomycetes, have revealed the metabolic flexibility of these groups, particularly in regard to their N cycling capabilities (Bentzon-Tilia, Severin & Hansen, 2015; Delmont et al., 2018; Martínez-Perez et al., 2018). Non-cyanobacterial diazotrophs are distributed throughout tropical and temperate latitudes and are sometimes the dominant members of diazotrophic communities (Bombar, Paerl & Riemann, 2016; Delmont et al., 2018; Langlois et al., 2015; Moisander et al., 2014). Notably, both non-cyanobacterial diazotrophs and UCYN have recently been identified as important constituents of temperate and coastal diazotroph communities (Bentzon-Tilia et al., 2015; Messer et al., 2015; Mulholland et al., 2012; Needoba et al., 2007; Rees, Gilbert & Kelly-Gerreyn, 2009; Shiozaki et al., 2015a; Short & Zehr, 2007), with their presence often associated with high rates of N_2_ fixation (Bentzon-Tilia et al., 2015; Tang et al., 2019).

In the coastal waters surrounding the Australian continent, bioavailable sources of N are regularly depleted (Condie & Dunn, 2006). Significant rates of N_2_ fixation have been observed throughout much of the tropical northern seas surrounding Australia (Bonnet et al., 2015; Messer et al., 2016; Messer et al., 2017; Montoya et al., 2004; Raes et al., 2014) and in the subtropical waters of the eastern Indian Ocean (Raes et al., 2015). High levels of diversity in *nifH* phylotypes have been detected throughout these regions, including important contributions by *Trichodesmium erythraeum*, UCYN-A, and the gammaproteobacterial group, Gamma A (Moisander et al., 2014; Bonnet et al., 2015; Messer et al., 2017). In contrast, our understanding of the importance of N_2_ fixation within the temperate waters along the southern coastline of Australia, which are dominated by large inverse estuaries, is severely limited.

Inverse estuaries represent unique ecosystems within the coastal zone of arid climates (Eyre, 1998), where an excess of evaporation over precipitation results in the formation of strong positive salinity gradients from marine at the mouth to hypersaline at the head (Nunes Vaz, Lennon & Bowers, 1990; Pritchard, 1952). In contrast to classical estuaries, inverse estuaries receive little to no freshwater input (Eyre, 1998; Smith & Veeh, 1989), and can become seasonally isolated from the adjacent continental shelf-waters when density fronts restrict oceanic inflow at the mouth (Petrusevics et al., 2011). Consequently, inverse estuaries can experience relatively oligotrophic conditions, giving rise to nutrient limitation in some systems (Middleton et al., 2013; Smith & Veeh, 1989).

Previously, we detected the presence of UCYN-A sub-lineages, UCYN-A1 and UCYN-A2, within the inverse estuary Spencer Gulf (Messer et al., 2015), an ecologically and economically important region of the south Australian marine environment (Deloitte Access Economics, 2017). Despite the fact that Spencer Gulf is typically oligotrophic, and primary production is reportedly N limited (Middleton et al., 2013), productive aquaculture industries and commercial fisheries are housed within the region, and the shallow waters act as foraging and nursery grounds for >30 species of threatened, protected, and iconic marine macro-organisms (Robbins et al., 2017). Seagrass-based N_2_ fixation has historically been suspected to be an important source of new N in the shallow upper region (Smith & Veeh, 1989), however, how pelagic productivity is maintained within the low-nutrient waters of Spencer Gulf remains an open question. To test the hypothesis that N_2_ fixation is a significant process within the temperate coastal waters of southern Australia, we investigated spatial and seasonal dynamics of N_2_ fixation activity, and diazotroph diversity, in Spencer Gulf and the surrounding shelf waters.

## Materials & Methods

### Sample collection

**Figure 1:**
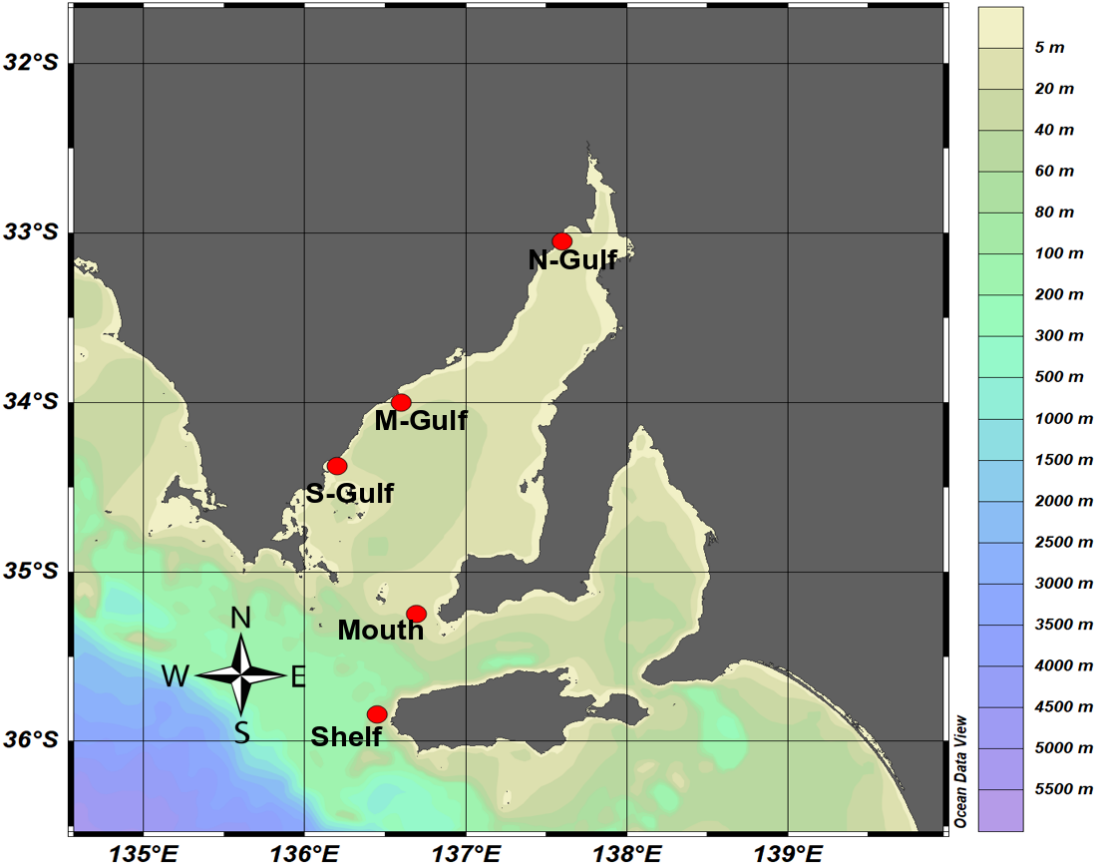
Sampling locations within Spencer Gulf and the adjacent continental shelf waters. Samples were collected from the Kangaroo Island National Reference Station (Shelf), Spencer Gulf mouth (Mouth), south western edge (S-Gulf), mid western edge (M-Gulf), and northern Spencer Gulf (N-Gulf), with ocean bathymetry shown as a colour chart generated using Ocean Data View (Schlitzer, 2018).

Surface seawater samples were collected from Spencer Gulf, a large inverse estuary within the South Australian Gulf System (∼22,000 km^2^), and from adjacent continental shelf waters. Spencer Gulf is characterised by steep gradients in sea surface temperatures and salinity and demonstrates marked differences in physicochemical characteristics during autumn/winter and spring/summer (Nunes & Lennon, 1986; Nunes Vaz, Lennon & Bowers, 1990; Petrusevics, 1993). Therefore, samples were collected during two contrasting seasons, in the Austral autumn between 28th April and 8th May 2014, and in the Austral summer between 2nd and 9th December 2014. Although considered oligotrophic, Spencer Gulf hosts productive aquaculture industries, which have been implicated in localised nutrient enrichment (Fernandes et al., 2007; Lauer et al., 2009). To capture local environmental variability, sampling was performed along a latitudinal gradient within the estuary, from an offshore site situated near Kangaroo Island [35.84S, 136.45E] on the continental shelf, through to the hypersaline region in the north of Spencer Gulf. Four locations inside Spencer Gulf were selected, including, Spencer Gulf mouth [35.25S, 136.69E] and three locations along the edge of the basin, southern Gulf [34.377S, 136.11E], mid-Gulf [33.92S, 136.58E] and northern Gulf [33.04S, 137.59E] (Fig. 1).

Sampling at the mouth and shelf sites were conducted from on-board the *RV Ngerin* in conjunction with routine monitoring for the Southern Australian node of the Integrated Marine Observing System (IMOS). Samples were collected at the Kangaroo Island National Reference Station (NRSKAI; referred to as “shelf” hereafter), and SAM8SG mooring locations (referred to as “mouth” hereafter) (Lynch et al., 2014). A shore-based sampling protocol was adopted for the southern, mid, and northern Gulf sampling sites, whereby surface samples were collected from jetties (piers), approximately 227, 154 and 440 m from the shore, respectively. In all cases, 60 L of water was collected from ∼1 m below the surface using a plastic bucket. Buckets and sample storage carboys were rinsed three times with sample water prior to filling and washed with 10% HCl and MilliQ between stations. The temperature and salinity of each sample was immediately recorded using a multi-parameter portable meter (WTW Profiline Multi 3320; Xylem Analytics, Germany).

### Dissolved inorganic nutrient analyses

To determine ambient concentrations of dissolved inorganic nutrients, including NO${}_{3}^{-}$ + NO${}_{2}^{-}$, PO${}_{4}^{3-}$ and SiO${}_{4}^{4-}$ (hereafter referred to as NO_3_/NO_2_, PO_4_, and SiO_4_ respectively), subsamples (45 ml) were collected in triplicate 50 ml Falcon tubes from each sampling site. Samples were immediately frozen at −20 °C and kept frozen prior to analysis. A Flow Injection Analyser (Lachat QuikChem 8000) was used to determine concentrations of NO_3_/NO_2_, PO_4_, SiO_4_ in the <0.45 µm filtrate from each thawed sample, with a limit of detection of 0.01 µM.

### Particulate carbon, nitrogen, and *δ*^**15**^N analyses

To provide an estimate of the concentrations of particulate carbon (C) and N in the planktonic material and the natural abundance of the ^15^N isotope in particulate matter (*δ* ^15^N, N_2_ fixation incubation T_0_), subsamples of between 2–4 L of surface seawater were collected from each site. Subsamples were filtered onto GF/F grade 0.7 µm filters (Whatman, Kent, UK) which were previously individually packaged in aluminium foil and pre-combusted at 450 °C for 4 h. Samples were stored double contained in two snap-lock bags and kept frozen at −20 °C, prior to being dried for 48 h at 60 °C. As previously described in Messer et al. (2017), filters were analysed on an elemental analyser (Thermo Finnigan MAT Conflo IV) coupled to an isotope ratio mass spectrometer (IRMS; Thermo Finnigan Delta XP; limit of detection = 15 µg N per filter) at the Research Corporation of the University of Hawaii.

### Biological N_**2**_ fixation incubations

To measure rates of N_2_ fixation activity among planktonic diazotrophs, we performed stable isotope tracer addition experiments at each site with ^15^N-labelled N_2_ gas. Acid-clean (10% HCl) 4 L Nalgene incubation bottles were rinsed three times with seawater from the site prior to being filled to over-flowing via silicone tubing, then capped with rubber septa head-space free. ^15^N_2_ gas (3 ml, 98 atom%, Sigma-Aldrich, Australia, lot SZ1670V, 2013 batch) was injected into each incubation bottle prior to inversion 100 times to disperse the gas bubble.

Samples for whole community N_2_ fixation (bulk seawater) and unicellular N_2_ fixation (<10 µm size fraction) were incubated in triplicate at *in situ* sea surface temperature using aquaria heaters and water circulation pumps attached to an outdoor 60 L plastic incubator, which was exposed to a natural diurnal light cycle at surface seawater intensity. ^15^N_2_ incubations were terminated via filtration after 24 h, by directly filtering the entire contents onto a pre-combusted (450 °C for 4 h; packaged in aluminium foil) GF/F grade 0.7 µm filter (Whatman; whole community), or through a 10 µm polycarbonate membrane filter (Isopore, EMD Merck Millipore, Billerica, MA, USA) onto a pre-combusted GF/F grade 0.7 µm filter (Whatman; unicellular size fraction). Enriched filters were stored double contained in two snap-lock bags to prevent any possible cross-contamination and kept frozen at −20 °C prior to analysis.

Following methods described in Messer et al. (2017), ^15^N_2_ amended GF/F filters were dried (60 °C for 48 h) separately to natural abundance samples to prevent cross-contamination. Total particulate N and C and isotopic composition were determined on an elemental analyser (Thermo Finnegan MAT Conflo IV) coupled to an IRMS (Thermo Finnegan Delta XP, limit of detection = 15 µg N per filter) at the Research Corporation of the University of Hawaii. Assimilation rates were calculated following Montoya et al. (1996). An atom% enrichment equivalent to 75% of the theoretical for a 24 h incubation was used as the enrichment factor for volumetric rate calculations to account for the incomplete dissolution of the ^15^N_2_ gas bubble (Großkopf et al., 2012; Mohr et al., 2010), following Messer et al. (2017).

### Collection, preservation, and extraction of microbial nucleic acids

In order to concentrate microbial cells for nucleic acid extraction, amplicon sequencing, and quantitative PCR, triplicate 2 L samples were filtered onto 0.2 µm membrane filters (Durapore, EMD Merck Millipore). Filters were stored at −20 °C during the field sampling (∼2 weeks) and transported to the laboratory on dry ice before being stored at −80 °C until extraction. The MoBio PowerWater DNA isolation kit (MoBio Laboratories, Carlsbad, CA, USA; now Qiagen) was used to extract microbial community DNA, following the manufacturer’s guidelines including an additional incubation with solution PW1 (10 min at 60 °C) prior to 10 min of bead beating, to ensure complete cell lysis.

### *nifH* amplicon sequencing and analyses

To determine the diversity of diazotrophic bacterioplankton, a fragment of the *nifH* gene was amplified using a nested protocol and the degenerate primers nifH3 and nifH4, and nifH1 and nifH2 (Zani et al., 2000; Zehr & Turner, 2001), largely following methods previously described (Messer et al., 2015; Messer et al., 2016; Messer et al., 2017). The following PCR reaction conditions were used to amplify the *nifH* gene: 95 °C (2 min) followed by 30 cycles of 95 °C (1 min), 48 °C (1 min) and 72 °C (1 min) followed by 72 °C (10 min). Amplification was confirmed using gel electrophoresis, replicates were pooled, and the resultant fragment was sequenced using the 454 FLX Titanium pyrosequencing platform (Roche, Nutley, NJ, USA) at Molecular Research LP (Shallowater, TX, USA).

The Quantitative Insights Into Microbial Ecology (QIIME) (Caporaso et al., 2010a; Caporaso et al., 2010b) open source software was used to process *nifH* pyrosequencing reads. Briefly, sequences were de-multiplexed and the low-quality sequences were removed (q <25 and <200 bp in length) using default parameters. Chimeric sequences were removed using USEARCH61 with default parameters against an unaligned version of a curated *nifH* reference database exported from Arb (downloaded from: http://wwwzehr.pmc.ucsc.edu/nifH_Database_Public/; Heller et al., 2014; Zehr et al., 2003). The remaining high-quality reads were clustered at 99% sequence identity using UCLUST, whereby sequences within 1% identity of the most abundant read were classified as operational taxonomic units (OTUs; Edgar, 2010). A representative sequence set was generated based on the most abundant sequence comprising an OTU. The PyNAST aligner tool (Caporaso et al., 2010a) was used with default parameters to BLAST and pairwise align representative *nifH* OTU sequences to those from the aligned version of the same curated *nifH* database used for chimera removal, providing putative taxonomy and “best hits” to primarily uncultured environmental sequences (Heller et al., 2014; Zehr et al., 2003). Any potential stop codons and frameshifts in the *nifH* sequences were identified using the FrameBot tool from the FunGene pipeline using default parameters (Fish et al., 2013). As part of this pipeline, taxonomy was assigned to the closest representatives within the Ribosomal Database Project’s *nifH* database based on amino acid identity (AAI) and sequence alignment (Fish et al., 2013). Finally, an OTU by sample matrix was generated, in which each sample was rarefied to the lowest number of sequences per sample (3,068) and singletons were removed prior to downstream analyses.

### Quantification of UCYN-A *nifH* genes

Based on our previous observations (Messer et al., 2015), we hypothesised that UCYN-A would be the most important diazotrophic group within Spencer Gulf and the adjacent continental shelf waters. In order to determine UCYN-A abundance, previously designed TaqMan qPCR probes (Table S1) were utilised to quantify the UCYN-A1 (Langlois, Hummer & LaRoche, 2008) and UCYN-A2 (Thompson et al., 2014) clades. qPCR standards were either cloned into the P-Gem T Easy Vector (Promega, Sydney, NSW, Australia) following the manufacturer’s guidelines (UCYN-A2) as previously described (Messer et al., 2017), or synthesised into the PUC-57 Amp (Genewiz) vector (UCYN-A1). The *nifH* gene inserts were then amplified from the plasmid DNA using plasmid specific PCR primers targeting the M13 binding site of the vector. A band of the correct size was purified from an electrophoresis gel using the Isolate II Gel/PCR Purification kit (Bioline, Eveleigh, NSW, Australia). DNA was then quantified using a Qubit Fluorometer and serially diluted to generate a standard curve incorporating 10^7^ to 10^1^ *nifH* copies.

qPCR reactions were performed as previously described in Messer et al. (2017). Specifically, template DNA was diluted 1:5 using nucleic-acid-free H_2_O to prevent inhibition and 5 µl of the template dilution was subsequently used in the qPCR assay. Each qPCR reaction included 200 nM of forward and reverse primer, 100 nM of TaqMan probe, 2x TaqMan Master Mix II, and 3 µl of nucleic-acid-free H_2_O. Samples were analysed in triplicate, with additional triplicate technical replicates and triplicate no template negative controls (5 µl nucleic-acid-free H_2_O), alongside the relevant standards (also analysed in triplicate). Reaction conditions were optimised for each primer and probe set using a combination of temperature, annealing time, and extension time gradients on a StepOnePlus™ Real-Time PCR machine (software v2.3; Applied Biosystems, Thermo Fisher Scientific, Scoresby, Victoria, Australia). The final optimal reaction conditions were identified to be: 50 °C (5 min), 95 °C (10 min) and 40 cycles of 95 °C (15 s) and 64 ° C (60 s) for UCYN-A2; and 95 °C (10 min) followed by 40 cycles of 95 °C (15 s), 55 °C (15 s) and 72 °C (60 s) for UCYN-A1. Linear regression analyses of quantification cycle (Cq) versus log10 *nifH* gene copies demonstrated that the UCYN-A2 assay had a mean R^2^ of 0.999 and an efficiency between 99.2–99.9% and the UCYN-A1 assay had a mean R^2^ of 0.993 and an efficiency between 92.0–98.7%. The Cq limit of detection and quantification for each assay was identified to be equivalent to ∼1–10 *nifH* copies per reaction.

### Statistical analyses

Prior to testing for significant differences between “season” and “site”, environmental data, N_2_ fixation rates, and qPCR data were checked for normality and homogeneity of variance using the Shapiro–Wilk and Brown-Forsythe tests respectively (SPSS, IMB Statistics 24). Data meeting these criteria were tested for significance using a one-way ANOVA, while a Kruskal Wallis ANOVA on ranks was used for data that failed to meet the stipulations of normality (SPSS, IMB Statistics 24). Pearson correlation coefficients and significance values were calculated (SPSS, IMB Statistics 24) between biological and environmental variable pairs across the entire dataset, and independently for samples collected in Austral autumn or summer.

Statistical analyses of diazotroph community dissimilarity were performed using the PRIMER 7 + PERMANOVA software. The final OTU by sample matrix was square-root transformed and a Bray Curtis resemblance matrix was generated. Significant differences between *nifH* amplicon sequencing profiles were explored using the non-parametric Analysis of Similarity (ANOSIM) test, using either “season” or “site” as a factor, while the contribution of each OTU to the observed dissimilarity between sampling sites was determined using Similarity Percentage analysis (SIMPER). In addition, a distance-based linear model (DistLM) was generated from the Bray-Curtis resemblance matrix, using the corresponding site-specific environmental metadata as predictor variables. Relationships between the environmental predictor variables and diazotroph community composition were also investigated using the BEST, biota and environment (BIOENV) test, using Spearman rank correlation.

The multivariate relationships between individual diazotroph OTUs, environmental metadata, and N_2_ fixation rates were explored using a negative binomial many-generalised linear model (Wang et al., 2012). The model was performed using the mvabund (v.4.1.3) package (Wang et al., 2012) in R (v4.0.2) and R studio (v1.3.959) (R Core Team, 2013). The *nifH* OTU by sample matrix was input as count data and converted to an mvabund object prior to model creation using the ‘manyglm’ function. The analysis of deviance table was generated using the ‘anova’ function with ‘p.uni = adjusted’ selected to correct for the effect of multiple testing.

## Results

### Environmental characteristics of Spencer Gulf and shelf waters

Consistent with the inverse estuarine nature and seasonal variability of Spencer Gulf, patterns in sea surface temperature (SST) and salinity exhibited a clear transition from cooler oceanic conditions in southern shelf waters, to warmer and hypersaline conditions in the northern region of the Gulf (Fig. 1; Table 1). Across this gradient, SST ranged from 18 °C to ∼23 °C, while salinity increased from 36 at the mouth to ≥ 40 at the northern site (Table 1). During the Austral autumn, SST was typically lower than SST observed during the summer (Table 1), with mean temperature (± standard deviation) across the five sites, 18.9 ± 0.8 °C relative to 21.0 ± 1.8 °C, respectively. In contrast, the salinity profile of Spencer Gulf was highly similar during both the Austral autumn and summer across the five sampling sites, with means for each season (± standard deviation) of 37.3 ± 1.65 and 37.1 ± 1.81, respectively.

**Table 1:** Physico-chemical metadata associated with each sampling site.

Sample	Sampling Time	Temp. (°C)	Salinity	NO_3_/NO_2_ (µM)	PO_4_ (µM)	SiO_4_ (µM)	PC (µg)	PN (µg)
A_Shelf	14:30	18.9	36.0	0.04	0.03	0.22	373	50.3
A_Mouth	8:00	18.7	36.0	0.02	0.06	0.36	377.8	53.2
A_S-Gulf	15:00	18	37.0	0.01	0.01	0.25	419.6	54.6
A_M-Gulf	7:30	18.8	37.7	0.01	0.02	0.52	341.7	37.8
A_N-Gulf	16:00	20.1	40.0	0.04	0.03	1.1	389.9	47.9
S_Shelf	6:30	18.7	36.0	0.02	0.08	0.24	1184.2	36.7
S_Mouth	9:00	19.6	36.0	0.01	0.04	0.24	762.7	44.6
S_S-Gulf	15:50	22.3	36.5	0.03	0.02	0.52	376.8	45.5
S_M-Gulf	7:55	21.1	36.9	0.03	0.05	0.53	755.5	62.8
S_N-Gulf	16:00	23.1	40.3	0.04	0.02	0.39	342.3	42.8

**Notes.**

A Autumn S Summer Temp. sea surface temperature PC particulate carbon PN particulate nitrogen

Sampling Time refers to the local time at the point of sample collection (Australian Eastern Standard Time).

Concentrations of dissolved inorganic nutrients were relatively stable between the southern shelf and northern Spencer Gulf waters. Indeed, NO_3_/NO_2_ concentrations were always <0.05 µM, and PO_4_ concentrations were generally low, ranging from 0.01 (i.e., limit of detection) to 0.08 µM across the five sampling locations (Table 1). Mean (± standard deviation) NO_3_/NO_2_ and PO_4_concentrations were similar between the two sampling periods, at 0.02 ± 0.01 and 0.03 ± 0.02 µM during Austral autumn, and 0.03 ± 0.01 and 0.04 ± 0.03 µM during Austral summer, respectively. Conversely, concentrations of SiO_4_ showed a sharp increase from the southern shelf to northern Gulf waters, ranging from 0.22 up to 1.10 µM (Table 1). While mean SiO_4_ concentrations were typically elevated during Austral autumn compared to summer, at 0.49 ± 0.36 and 0.38 ± 0.14 µM, respectively.

### Biological N_**2**_ fixation rates in temperate southern Australia

**Figure 2:**
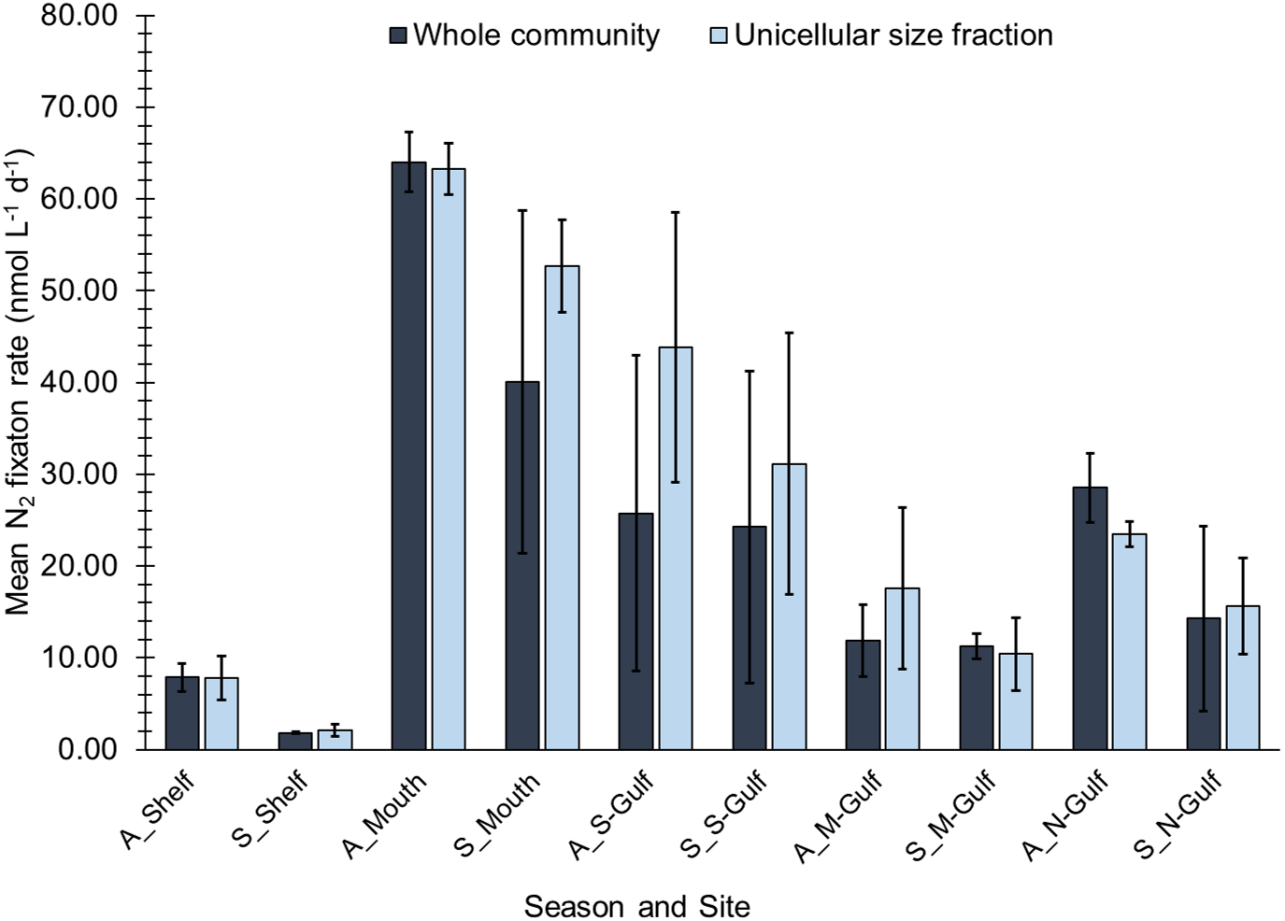
Biological *N*_2_ fixation rates measured during Austral autumn and summer in south Australian coastal waters. Rates have been corrected to account for the incomplete dissolution of the ^15^*N*_2_ gas bubble (see Methods). Error bars represent the standard deviation about the mean (*n* = 3).

Measurable rates of N_2_ fixation occurred at all sites during both the Austral autumn and summer, but rates were highly heterogeneous ranging from 2 nmol L^−1^ d^−1^to 64 nmol L^−1^ d^−1^ (Fig. 2). Across the entire dataset, no significant differences were observed between whole community (WC) and unicellular size fraction (USF) N_2_ fixation rates (Kruskal-Wallis test, *H* = 0.32, d.f. = 1, *n* = 30, *P* = 0.574), indicating that the unicellular size fraction contributed the majority of the observed N_2_ fixation activity. Overall, no significant differences in N_2_ fixation rates were observed between incubations conducted during Austral autumn compared to summer (Kruskal-Wallis test, *H* = 1.397 and 1.931, d.f. = 1, *P* = 0.237 and 0.165, for WC and USF respectively; *n* = 15 per season). During both Austral autumn and summer, WC and USF N_2_ fixation rates were highly correlated, with Pearson correlation coefficients (r) of 0.85 and 0.76 respectively, further supporting the proposition that the unicellular size fraction contributed the majority of the observed N_2_ fixation activity.

When grouped by “site” as opposed to “season”, N_2_ fixation rates exhibited significant spatial heterogeneity (One-way ANOVA, *P* ≤ 0.001, *F* = 37.38, d.f. = 4, *n* = 6 per site). The lowest rates of N_2_ fixation during both the Austral autumn and summer occurred in the southern shelf waters, with maximum rates at this site reaching only 8 ± 2 nmol L^−1^ d^−1^(mean ± standard deviation; Fig. 2). In contrast, N_2_ fixation rates peaked in the waters at the mouth of Spencer Gulf, where they reached 64 ± 3 and 40 ± 19 nmol L^−1^ d^−1^, in Austral autumn and summer respectively (Fig. 2). Relative to rates observed at the mouth of Spencer Gulf, N_2_ fixation rates decreased at the southern and mid-western sites of the gulf during both autumn and summer (Fig. 2). N_2_ fixation rates then showed a notable increase at the northern-gulf site, reaching 29 ± 4 nmol L^−1^ d^−1^ during Austral autumn, and 14 ± 10 nmol L^−1^ d^−1^ during Austral summer (Fig. 2).

N_2_ fixation rates were significantly correlated with low concentrations of NO_3_/NO_2_ (Pearson’s r: -0.53; *P* = 0.002, *n* = 30, USF). This relationship was maintained when considering only Austral summer samples (r: -0.64; *P* = 0.01; *n* = 15, USF), but not when only considering those collected during Austral autumn. In contrast, during the Austral autumn N_2_ fixation rates were positively correlated to PO_4_ concentrations (r: 0.73; *P* = 0.002; *n* = 15, WC). No significant relationships were observed between N_2_ fixation and SST or salinity, despite clear spatial gradients in these environmental parameters (Table 1).

### Diversity and composition of *nifH* containing bacterioplankton

After rarefaction to 3,068 sequences per sample and the removal of singletons, between 159 and 332 *nifH* OTUs were detected at each sampling site. The diversity of *nifH* containing bacterioplankton increased along the latitudinal gradient of Spencer Gulf, whereby Shannon’s Diversity (H’) was lowest in the southern shelf waters, where H’ = 1.95 and 2.86 and peaked at the mid-western edge of Spencer Gulf, where H’ = 4.97 and 4.37, during Austral autumn and summer respectively (Table S2). Despite the site-specific differences in diazotroph diversity, mean H’ across the Gulf was approximately equal for both sampling seasons, whereby H’ = 3.67 during austral autumn, and H’ = 3.58 during austral summer.

**Figure 3:**
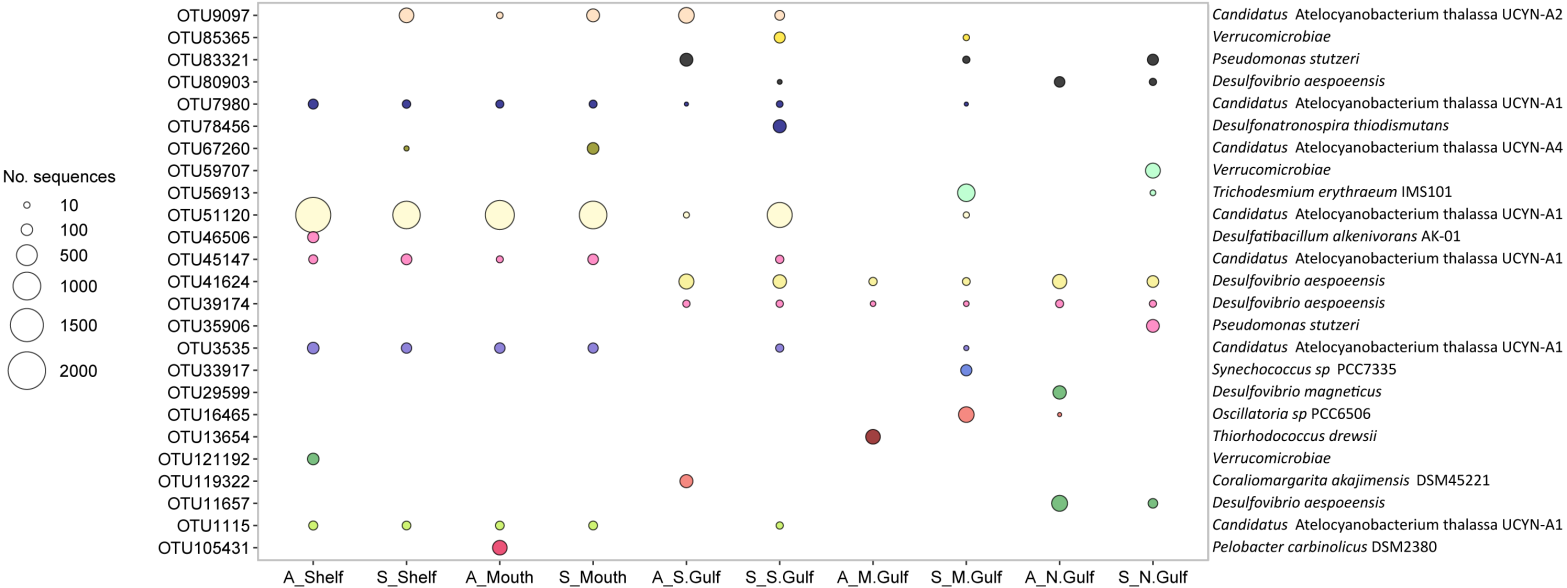
Relative abundance of the top 25 *nifH* OTUs and their taxonomic assignment (closest representative) detected within south Australian coastal waters during Austral autumn (A_) and summer (S_).

Phylogenetic analyses of *nifH* sequences demonstrated that the most abundant OTUs (*n* = 25), equivalent to ∼53% of total sequences and between 15 and 82% of sequences for any given sample, comprised a mixture of Cluster 1 and Cluster 3 diazotrophs at ≥ 83% amino acid identity (AAI; Table S3). A Bray-Curtis resemblance matrix of rarefied *nifH* sequence data was used to compare diazotroph community composition within and between the southern shelf waters and Spencer Gulf sampling locations, revealing significant spatial variability in diazotroph assemblage structure (ANOSIM, R: 0.59, *P* = 0.005). SIMPER analysis revealed 99.7% and 100% community dissimilarity between northern Gulf diazotroph assemblages and those in the shelf waters and at the mouth of the Gulf, respectively. Diazotroph assemblages in the shelf waters and mouth were dominated by five OTUs identified to be the UCYN-A1 open ocean ecotype (OTU51120, OTU3535, OTU45147, OTU7980, and OTU1115; Fig. S1), which collectively represented 75% and 56% of sequences at the shelf during Austral autumn and summer respectively (Fig. 3). Similarly, these OTUs comprised 54% and 58% of sequences at the mouth during Austral autumn and summer (Fig. 3). Correspondingly, diazotroph communities in the southern shelf waters and at the mouth of the gulf shared the greatest similarity in composition, with SIMPER analysis revealing only 63.4% dissimilarity between these populations. The dissimilarity between the shelf waters and the mouth was largely driven by the coastal and open ocean ecotypes UCYN-A2 and UCYN-A4 (OTU9097 and OTU67260, respectively), which were collectively present at higher relative abundances at the mouth (Fig. 3).

Spencer Gulf communities showed a decline in the abundance of UCYN-A OTUs, and a greater proportion of sequences associated with non-cyanobacterial diazotrophs, along with a small proportion of OTUs closely related to filamentous cyanobacteria such as *Trichodesmium erythraeum* (Fig. 3; Table S3). The average relative abundance of two UCYN-A1 open ocean group OTUs, (OTU51120 and OTU3535), were identified by SIMPER analysis as the main drivers of community dissimilarity between the shelf waters, Spencer Gulf mouth, and northern gulf diazotroph assemblages. At the southern gulf site, a transitional community was observed, which comprised UCYN-A1 and UCYN-A2 (12–44% of sequences), *Pseudomonas stutzeri* (7%), *Desulfovibrio aespoeensis* (8–12%), *Coraliomargarita akajimensis* (7%), and *Desulfonatronospira thiodismutans* (7%). In contrast, at the northern site the community was primarily comprised of OTUs related to *Desulfovibrio aespoeensis* (10–28%), *Pseudomonas stutzeri* (11%), and Verrucomicrobiae (11%; Fig. 3). SIMPER analysis identified the *Desulfovibrio aespoeensis* OTU (OTU41624; 96% AAI similarity) as also being responsible for the between-site discrimination of the diazotroph community, with this OTU absent from assemblages detected in the southern waters. Interestingly, only a small proportion of the most abundant 25 OTUs were represented at the mid-Spencer Gulf site (15–38%) and northern-Spencer Gulf site (32–36%) especially during the Austral autumn. Instead, overall low abundance OTUs, which were typically unique to these sites (i.e., OTUs representing <0.5% of total sequences), were responsible for the high alpha diversity associated with these sites.

Across the dataset, several variables were identified as having a significant effect on the relative abundance and composition of diazotrophic bacterioplankton within Spencer Gulf and the adjacent shelf waters. These included NO_3_/NO_2_ (*P* = 0.002), N_2_ fixation by the unicellular size fraction (*P* = 0.007) and the whole community (*P* = 0.016), salinity (*P* = 0.017), temperature (*P* = 0.037), particulate nitrogen (PN; *P* = 0.039), and PO_4_(*P* = 0.048; Many GLM, Table S4). Only three of these predictors displayed significant relationships (adjusted *P*-value <0.1) with individual OTUs, including PN (1 OTU), N_2_ fixation by the unicellular size fraction (14 OTUs), and salinity (4 OTUs; Table S4).

Approximately 33% of the spatial variation in diazotroph community dissimilarity could be explained by ambient salinity and SiO_4_ concentrations (DistLM R^2^: 0.33; salinity *F* = 2.24, *P* = 0.001; SiO_4_ *F* = 1.56, *P* = 0.028; *n* = 10). The importance of salinity and SiO_4_ in structuring the diazotroph community was further confirmed by BEST/BIOENV analyses, resulting in a significant (*P* = 0.01, *n* = 10) coefficient, Rho = 0.67, using Spearman’s Rank correlation. The sequential addition of the environmental parameters, PN, NO_3_/NO_2_, and PO_4_, reduced the strength of the correlation to 0.56, 0.52, and 0.49, respectively. In contrast to the observed spatial heterogeneity in diazotroph assemblage structure, no significant differences in diazotroph community dissimilarity were observed between the Austral autumn and summer sampling times (ANOSIM, R: −0.12, *P* = 0.80).

### Abundance of UCYN-A1 and UCYN-A2 *nifH* genes

**Figure 4:**
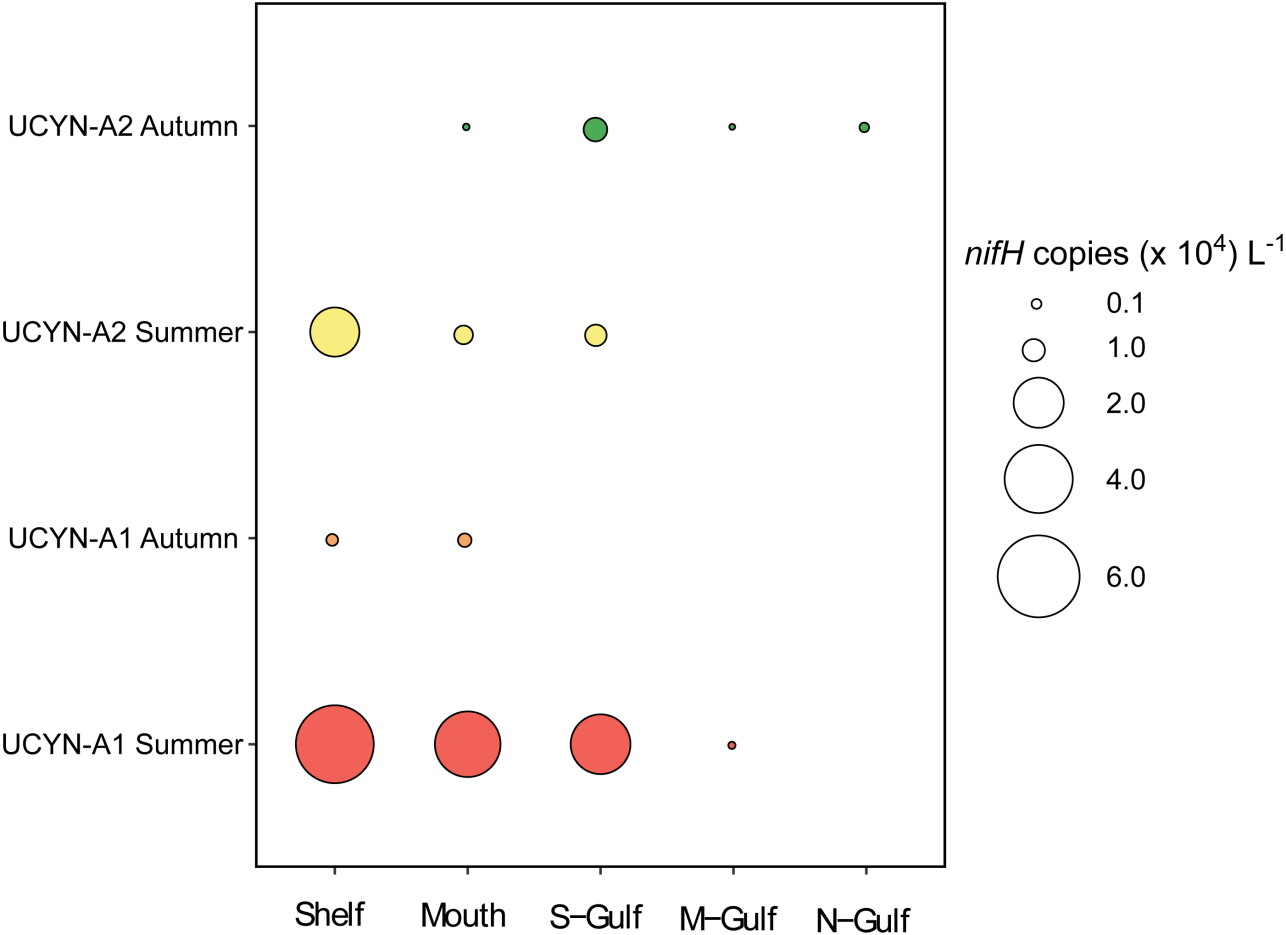
Mean qPCR derived abundances of UCYN-A1 and UCYN-A2 in south Australian coastal waters (*n* = 3).

qPCR derived abundances of UCYN-A1 and UCYN-A2 *nifH* genes demonstrated higher abundances of these organisms in shelf waters and at the more southern sites of Spencer Gulf (Fig. 4). Specifically, the maximum mean abundance of UCYN-A1 occurred in the southern shelf waters during Austral summer, whereby 5.4 ± 4.7 × 10^4^ *nifH* copies L^−1^ were detected (Fig. 4). Similarly, UCYN-A2 also reached maximum abundance in the shelf waters during Austral summer, with mean *nifH* copies 1.9 ± 1.4 × 10^4^ L^−1^ (Fig. 4).

Across all sampling locations, UCYN-A1 was significantly more abundant during the Austral summer compared to autumn (Mann Whitney test, U: 69, *P* < 0.05). While UCYN-A2 abundances did not differ significantly between the Austral autumn and summer sampling.

Across the entire dataset, UCYN-A1 abundance was positively correlated with concentrations of PO_4_ (r: 0.39; *P* = 0.03, *n* = 30). In contrast, overall UCYN-A2 abundance was not significantly correlated with any of the measured environmental parameters. However, when analysed by “season”, UCYN-A2 abundance was negatively correlated to SST during both Austral autumn and summer (*n* = 15 per season; r: −0.55 and r: −0.53, *P* = 0.03 and 0.04, respectively). In addition, during Austral autumn UCYN-A2 abundance was negatively correlated to PO_4_ concentrations (r: −0.53; *P* = 0.04; *n* = 15). Despite the potential importance of salinity in structuring the overall diazotroph community, no significant relationships were observed between UCYN-A qPCR derived abundances and salinity.

## Discussion

Increasing evidence suggests that temperate coastal waters may be overlooked hotspots of N_2_ fixation activity (Mulholland et al., 2012; Mulholland et al., 2019; Tang et al., 2019). Determining the distribution and activity of diazotrophs, and the environmental processes that influence them within coastal zones is therefore important to further our understanding of N availability across diverse marine environments. Compared to previous studies in temperate and tropical estuarine environments, where maximum N_2_ fixation rates of 30–85 nmol L^−1^ d^−1^ have been observed (Ahmed et al., 2019; Bentzon-Tilia et al., 2015; Bhavya et al., 2016), here we report relatively high rates of N_2_ fixation in temperate coastal waters of southern Australia within the inverse estuary Spencer Gulf. We show that N_2_ fixation rates, diazotroph diversity, and community structure, can vary considerably across relatively small spatial scales, however the dynamics of N_2_ fixation were relatively stable across two contrasting seasons. Our findings suggest that N_2_ fixation, possibly mediated by UCYN-A and non-cyanobacterial diazotrophs, may provide an important source of fixed N to support primary production within the oligotrophic, temperate coastal waters of southern Australia.

### N_**2**_ fixation in temperate coastal environments

Recent efforts to determine the importance of N_2_ fixation as a source of new N within temperate coastal waters have revealed N_2_ fixation activity in these regions is similar to, and at times higher than, rates reported for tropical and subtropical open ocean environments (Mulholland et al., 2019; Tang et al., 2019). For example, maximum N_2_ fixation rates of 65, 130, and 100 nmol L^−1^ d^−1^ have recently been observed in coastal waters of the north-eastern, mid-, and western Atlantic Ocean respectively (Fonseca-Batista et al., 2019; Mulholland et al., 2019; Tang et al., 2019). In environments representing the traditional niche of N_2_ fixation, such as the North Pacific Subtropical Gyre (NPSG) and the Eastern South Pacific (ESP), maximum N_2_ fixation rates have been reported to be considerably lower at ≤ 20 nmol L^−1^ d^−1^ (Böttjer et al., 2017; Gradoville et al., 2017; Shiozaki et al., 2017).

In the temperate coastal waters of southern Australia, we observed relatively high rates of N_2_ fixation, with a maximum N_2_ fixation rate of 64 nmol L^−1^ d^−1^. This observation is similar in magnitude to the high N_2_ fixation rates reported for the tropical oligotrophic seas of northern Australia (Bonnet et al., 2015; Messer et al., 2016), and is almost double maximum N_2_ fixation rates previously reported for tropical estuarine systems (31–34 nmol L^−1^ d^−1^; Ahmed et al., 2019; Bhavya et al., 2016). The lowest rates of N_2_ fixation (2 nmol L^−1^ d^−1^) occurred in the continental shelf waters. This finding is comparable to observations from other continental shelf ecosystems where N_2_ fixation rates are typically lower that those observed in sites closer to the coast (Mulholland et al., 2012; Shiozaki et al., 2015a; Singh, Gandhi & Ramesh, 2019). Intermediate rates of N_2_ fixation (10–45 nmol L^−1^ d^−1^) were measured within Spencer Gulf, and these rates are placed within the upper end of those previously reported for other temperate coastal, and tropical estuarine waters (Ahmed et al., 2019; Bentzon-Tilia et al., 2015; Bhavya et al., 2016; Mulholland et al., 2012; Rees, Gilbert & Kelly-Gerreyn, 2009; Shiozaki et al., 2015a). Importantly, our findings demonstrate that N_2_ fixation in the temperate waters of southern Australia are similar to, and can exceed, those observed in the NPSG and ESP (Gradoville et al., 2017).

While N_2_ fixation rates demonstrated clear spatial patterns in their magnitude between southern shelf and Spencer Gulf waters, we observed relatively consistent N_2_ fixation rates across opposing seasons. This is in contrast to previous seasonal observations of N_2_ fixation from distinct marine environments, where N_2_ fixation rates are typically higher during spring/summer than autumn/winter and are accompanied by shifts in the abundance of different diazotrophic taxa (Bentzon-Tilia et al., 2015; Böttjer et al., 2017; Fernandez et al., 2015; Mulholland et al., 2019). We hypothesised that seasonal differences in N_2_ fixation rates would occur within Spencer Gulf and shelf waters due to the known seasonality in physico-chemical characteristics, such as temperature, salinity, and dissolved nutrients, which ultimately influence the distribution and activity of marine diazotrophic microorganisms (Monteiro, Dutkiewicz & Follows, 2011; Moore et al., 2013; Ward et al., 2013). However, while limited in replication, we observed relatively stable site-specific physico-chemical conditions between the two contrasting seasons, and no significant differences in the composition of the underlying diazotrophic community. While limited in scope to two time-points, our observations suggest that relatively high N_2_ fixation rates can be maintained within Spencer Gulf while favourable conditions prevail. In future, increased sampling resolution is required to define the seasonal dynamics of N_2_ fixation within the temperate coastal waters of southern Australia.

### Regional significance of biological N_**2**_ fixation

Our previous research indicated that the pelagic microbial community of Spencer Gulf includes a diverse array of diazotrophic clades (Messer et al., 2015). However, the presence of diazotrophic groups cannot solely be used as evidence for the importance of pelagic N_2_ fixation, as the physiological process is tightly regulated (Paerl, Crocker & Prufert, 1987). To the best of our knowledge, our observations of N_2_ fixation within the pelagic realm of Spencer Gulf represent the first N_2_ fixation measurements from a temperate inverse estuary. Our N_2_ fixation rate measurements support our hypothesis that pelagic N_2_ fixation may provide a supply of fixed N within Spencer Gulf and the southern shelf waters, at considerably high rates relative to tropical and subtropical open ocean environments. In an earlier study, Middleton et al. (2013) estimated the influx of bioavailable N (in the form of NO_3_ and NH_4_) within Spencer Gulf to be 16.9 kilotonnes yr^−1^, including anthropogenic N sources and mixing of upwelled nutrients from continental shelf waters. This estimate did not include biological N_2_ fixation as a source of N, using their estimate of the volume of Spencer Gulf (4.58 ×10^14^ L), the N_2_ fixation rates measured herein could theoretically contribute an additional 23–149 kilotonnes N yr^−1^, albeit assuming consistent daily N_2_ fixation rates for a given site. Indeed, an accurate N budget would require extensive additional N_2_ fixation rates, with the appropriate modifications to the bubble method used to measure N_2_ fixation (White et al., 2020). Nevertheless, based on our estimates, we propose that the process of biological N_2_ fixation could be one mechanism by which productivity is maintained throughout the region.

It must be noted that the N_2_ fixation rates presented herein have been corrected to allow for the incomplete dissolution of the ^15^N_2_ gas bubble at 75% of the theoretical for a 24 h incubation (Großkopf et al., 2012; Mohr et al., 2010). However, recent methodological comparisons suggest no “global factor” exists for rate corrections to the bubble method (Wannicke et al., 2018; White et al., 2020). Despite the known caveats of the bubble method, this approach was used in the present study due to the predicted highly dissimilar environmental conditions at each site, which would be very difficult to replicate with pre-prepared ^15^N_2_ saturated artificial seawater (Wilson et al., 2012). In particular, the observed gradient in ambient temperature and salinity, which determines gas solubility and is accounted for in the rate calculations based on our observations at each site, would be difficult to anticipate ahead of sample collection. We also note that contamination of Sigma-Aldrich commercial ^15^N_2_ gas stocks was reported after our initial study (Dabundo et al., 2014). Although we cannot explicitly rule out contamination in the batch of ^15^N_2_ that we used, assuming the mean values for Sigma Aldrich lot SZ1670V reported in Table 1 of Dabundo et al. (2014) are consistent across batches, we estimate that potential contamination from ^15^NO_3_, ^15^NH_4_, and ^15^N_2_O, would represent an extremely small proportion of additional ^15^N in our incubations, equivalent to a total of 3.2 x 10^−7^ moles. In our experiments, the relative concentration of ^15^N gas added was 2.7 × 10^−4^ moles. Including this estimate of additional ^15^N in our trace additions, any potential contamination would inflate our N_2_ fixation rates by between 0.001–0.079 nmol L^−1^ d^−1^, which is within the lower end of the inferred N_2_ fixation rates resulting from ^15^NH_4_ contamination for 4.5 L incubations, presented in Table 2 of Dabundo et al. (2014)). Moreover, this estimate is within our calculated standard error of mean N_2_ fixation rates across triplicate samples (equivalent to 0.05 - 10.77 nmol^−1^ d^−1^). Therefore, any potential contamination would have a negligible effect on the ultimate N_2_ fixation rates reported herein. In future work, the modified bubble method should be employed, including additional determination of ^15^N_2_ atom% enrichment of individual incubation bottles and ^15^N_2_ gas purity, as recently suggested by the scientific community (Jayakumar et al., 2017; Klawonn et al., 2015; White et al., 2020).

### Identifying the key players in coastal N_**2**_ fixation

Understanding the abundance and composition of the diazotrophic community underlying N_2_ fixation activity is important for deciphering the potential impact of newly fixed N to a given region (Mulholland, 2007; Zehr & Kudela, 2011). For instance, throughout tropical and subtropical open ocean environments, N_2_ fixation by autotrophic diazotrophs such as *Trichodesmium* sp. will contribute directly to local primary production and may also release recently fixed N_2_ into the water column to support the growth of non-diazotrophic organisms (Berthelot et al., 2015; Caffin et al., 2018; Garcia, Raimbault & Sandroni, 2007; Glibert & Bronk, 1994; Mulholland, Bronk & Capone, 2004). On the other hand, symbiotic diazotrophs such as the heterocystous cyanobacterium *Richelia*, which is typically associated with “tropical” phytoplankton species, transfer fixed N_2_ directly to their eukaryotic phytoplankton host (Foster et al., 2011), and therefore contribute to new production and carbon sequestration in regions where they are abundant, such as the NPSG (Karl et al., 2012). While the contribution of newly fixed N by non-cyanobacterial diazotrophs is not yet clear (Turk-Kubo et al., 2014), their combined high abundances and widespread transcriptional activity in areas of high N_2_ fixation rates (Bird & Wyman, 2013; Chen et al., 2018; Langlois et al., 2015; Moisander et al., 2014), indicate that they could make an important contribution to support primary production in both open ocean and coastal environments.

The diversity of diazotrophic organisms detected in the present study indicates that N_2_ fixation activity may directly and indirectly support primary production within Spencer Gulf. Within the diverse diazotrophic communities detected, Cluster 1B UCYN-A, and Cluster 1G and Cluster 3 Proteobacteria, dominated diazotroph community profiles. Specifically, we observed high relative abundances of sequences closely related (≥ 96% AAI) to the symbiotic UCYN-A, in addition to the presumed free-living *Pseudomonas stutzeri* and *Desulfovibrio aespoeensis*, as well as lower relative abundances of the large filamentous tropical cyanobacterium *Trichodesmium erythraeum*. To date, UCYN-A and gammaproteobacterial diazotrophs (related to *Pseudomonas stutzeri*), have consistently been observed within temperate coastal diazotroph communities (Bentzon-Tilia et al., 2015; Mulholland et al., 2019; Needoba et al., 2007; Shiozaki et al., 2015b), but they are also key components of subtropical and tropical assemblages (Bonnet et al., 2015; Langlois et al., 2015; Moisander et al., 2014). Our observations provide further support for the global significance of these groups, although it must be noted that the *Pseudomonas stutzeri* OTUs did not cluster with known sequences from the globally distributed Gamma A clade.

While the presence of *Trichodesmium erythraeum* was somewhat unexpected due to its tropical and subtropical distribution (Capone et al., 2005), sequences related to *Trichodesmium* sp. have previously been observed at temperate latitudes of the Atlantic and Pacific Oceans (Mulholland et al., 2019; Rivero-Calle et al., 2016; Shiozaki et al., 2015a), and their presence has also been reported in south Australian waters based on microscopic observations (Paxinos, 2007). We did not determine the specific activity of *Trichodesmium sp*. within our samples, however, it comprised up to 17% of the diazotroph community at the mid-Gulf site during Austral summer. Owing to its presence and potential importance for both local primary production and N availability, further investigation into the significance of *Trichodesmium sp.* within temperate coastal waters is required.

Consistent with our previous observations of UCYN-A diversity and distribution within Spencer Gulf (Messer et al., 2015), we observed differences in the abundances of the open-ocean UCYN-A1 and the coastal UCYN-A2 within and between the southern shelf waters. The emerging sub-lineage UCYN-A4 (Farnelid et al., 2016) was also detected in our amplicon sequencing profiles during Austral summer. Due to the similarity between this OTU and the UCYN-A2 qPCR assay of Thompson et al. (2014), we cannot rule out that our qPCR derived abundances do not contain a mixture of the UCYN-A2 and UCYN-A4 sub-lineages (Farnelid et al., 2016). Since UCYN-A1, and to a lesser extent UCYN-A2 (possibly A2/A3/A4 sub-lineages), have recently been shown to be highly abundant (≤ 10^6^ *nifH* copies L^−1^) and reasonably active, fixing N_2_ at rates of 6 nmol L^−1^ d^−1^ in the cold surface waters of the Western Arctic Ocean (Harding et al., 2018), UCYN-A are highly likely to be important mediators of N_2_ fixation within Spencer Gulf and more broadly across temperate and coastal marine environments.

### What environmental factors influence N_**2**_ fixation in temperate southern Australian waters?

Across the global ocean, SST and subsurface minimum dissolved oxygen concentrations have been identified as the major environmental variables influencing pelagic N_2_ fixation rates (Luo et al., 2014; Tang, Li & Cassar, 2019). In addition, the availability of dissolved iron, phosphorus, other N sources (Landolfi et al., 2015; Ward et al., 2013), and grazing by zooplankton (Wang et al., 2019), have all been identified as factors shaping the distribution and magnitude of marine N_2_ fixation. Although limited in scope and replication, in the temperate southern Australian waters examined here, high N_2_ fixation rates during the Austral autumn were significantly correlated with increased PO_4_ concentrations, as was the overall abundance of UCYN-A1 (derived by qPCR). This is consistent with patterns observed in other temperate coastal waters, where N_2_ fixation has previously been shown to be significantly correlated with phosphorus availability (Tang et al., 2019). This pattern is also in-line with patterns observed within more oceanic waters, where PO_4_ availability has been shown to influence *nifH* expression and N_2_ fixation rates in experimentally manipulated and natural diazotroph assemblages (Rees, Law & Woodward, 2006; Sañudo Wilhelmy et al., 2001; Turk-Kubo et al., 2012; Watkins-Brandt et al., 2011). As phosphorus is an important constituent of cellular and molecular machinery, there is likely a direct causal relationship between PO_4_ and N_2_ fixation, whereby diazotroph abundances and N_2_ fixation rates are increased under P-replete conditions, as has previously been observed for the UCYN-A1-haptophyte symbiosis (Krupke et al., 2015). Within Spencer Gulf, phytoplankton growth is estimated to be limited by PO_4_ availability year-round (Middleton et al., 2013), indicating that while higher N_2_ fixation rates may provide a source of bioavailable N to the dissolved pool, the increased diazotrophic activity may deplete PO_4_ concentrations for non-diazotrophic microorganisms.

In the present study, overall N_2_ fixation rates were also negatively correlated with concentrations of NO_3_/NO_2_, which are typically depleted in Gulf waters during Austral summer yet may remain relatively high on the continental shelf due to a permanent deep nutrient pool (Doubell et al., 2018). Spencer Gulf and the adjacent continental shelf waters are characterised by a unique combination of oceanographic and regional circulation processes that create seasonal and localised east–west gradients in ambient concentrations of key macro- and micro-nutrients, underpinning variability in microbial productivity (Doubell et al., 2018; Middleton et al., 2013; Van Ruth et al., 2018). During Austral autumn, the density front at the entrance to Spencer Gulf begins to break down and an influx of continental shelf water, relatively rich in macronutrients, enters the Gulf along the western edge, while the oligotrophic Gulf water exits from the eastern side of the mouth (Middleton & Bye, 2007). These north-south and east–west gradients in NO_3_/NO_2_ and PO_4_ concentrations (low to relatively high, respectively) (Middleton et al., 2013), may explain the observed correlations between N_2_ fixation rates and these nutrients. This suggests that increased N_2_ fixation activity may occur due to the low concentrations of bioavailable N, further indicating that N derived from N_2_ fixation could sustain productivity within the N limited Spencer Gulf region. Recently, N_2_ fixation by UCYN-A was shown to occur even when dissolved inorganic nitrogen sources are replete, and may even be stimulated by increased NO_3_ concentrations (Mills et al., 2020), highlighting the complexity of factors governing N_2_ fixation activity in the environment. Collectively, our observations in fact represent the classic nutrient regime within which diazotrophs gain a competitive advantage over non-diazotrophic microorganisms (Ward et al., 2013), utilising excess PO_4_ and fixing N_2_ to support growth.

While PO_4_ and NO_3_/NO_2_ were correlated with rates of N_2_ fixation at the sites examined in this study, they were not significant predictors of diazotroph assemblage structure. Rather, the structure of the underlying diazotroph community was significantly influenced by the prevailing salinity and SiO_4_ concentrations. Regional variability in SiO_4_ concentrations may reflect abiotic indicators of different water masses, and may drive distinct differences in the composition of microbial assemblages (Foster et al., 2007; Weber et al., 2017). The observed transition towards increased non-cyanobacterial diazotrophs in the upper shallow waters of the Gulf could be indicative of their redistribution from the sediment or seagrass microbiome (Brown, Friez & Lovell, 2003; Lehnen et al., 2016), and warrants further exploration of their specific activity, source and contribution to N cycling in Spencer Gulf.

Salinity is a major structuring factor for estuarine microbial communities, driving the transition from freshwater- to marine-adapted lineages (Bouvier & Del Giorgio, 2002; Jeffries et al., 2016; Kirchman et al., 2005), and influencing rates of biogeochemical nutrient cycling (Bernhard et al., 2007; Bhavya et al., 2016). Unlike classical estuaries, inverse estuaries such as Spencer Gulf experience hypersaline conditions at the head of the estuary and marine salinities at the mouth, which has previously been shown to influence the overall composition of specific cyanobacterial ecotypes (Messer et al., 2015). In the present study, hypersaline regions of Spencer Gulf were associated with an increase in the relative abundance of non-cyanobacterial diazotrophs and a decrease in the abundance of UCYN-A at sites with salinities >∼37 PSU, which may reflect an inhibitory effect of high salinity on UCYN-A and it’s eukaryotic host. In contrast, members of the deltaproteobacteria, related to the Cluster 3 diazotrophs observed in the present study, have previously been shown to be moderately halophilic (Gam et al., 2009; Warthmann et al., 2005), and their increased relative abundances at the northern most stations of Spencer Gulf suggests they are likely to be halotolerant.

## Conclusions

This study provides further evidence that marine N_2_ fixation is not limited to tropical and subtropical open ocean environments, yet is widespread throughout diverse, temperate ecosystems, which have previously been overlooked as hotspots of N_2_ fixation activity. Our results indicate that N_2_ fixation is influenced by an interplay of physical and chemical environmental variables, which may have direct and indirect effects on the distribution and activity of diazotrophs in coastal waters. Our data revealed notable stability in N_2_ fixation across contrasting seasons, suggesting that the oligotrophic conditions of southern Australian coastal waters promote diazotrophy within the region. Notably, our findings suggest that pelagic N_2_ fixation, mediated by UCYN and non-cyanobacterial diazotrophs, could provide a greater source of fixed N than upwelled and anthropogenic bioavailable N within the coastal waters of southern Australia.

##  Supplemental Information

10.7717/peerj.10809/supp-1 Table S1 TaqMan qPCR primer and probe sequences used to quantify UCYN-A1 and UCYN-A2 *nifH* copies

10.7717/peerj.10809/supp-2 Table S2 Diversity (H’) of diazotrophic communities within south Australian coastal waters

10.7717/peerj.10809/supp-3 Table S3 Phylogenetic affiliation of the most abundant *nifH* OTUs (25 OTUs, equivalent to 50% of total sequences) *nifH* subclusters, and the closest cultured representatives, were determined by the FunGene pipeline. AAI = amino acid identity.

10.7717/peerj.10809/supp-4 Table S4 Analysis of Variance Table results from the many GLM Multivariate Model Contains the output from the many generalised linear model, including the mutivariate and univariate test statistics and adjusted P-values.

10.7717/peerj.10809/supp-5 Figure S1 Maximum likelihood subtree of *nifH* OTUs identified as “Candidatus Atelocyanobacterium thalassa” Tree inference was performed using the Maximum Likelihood method and Tamura-Nai model in MEGAX (v10.1.8; Kumar et al., 2018. The scale bar represents the number of substitutions per site.
Kumar S., Stecher G., Li M., Knyaz C., and Tamura K. (2018). MEGA X: Molecular Evolutionary Genetics Analysis across computing platforms. Molecular Biology and Evolution 35:1547-1549.

10.7717/peerj.10809/supp-6 Supplemental Information 6 N2 fixation raw and rate calculation data Includes the raw particulate nitrogen and carbon, and stable isotope data for each sample, including the T0 natural abundance and T24 incubation data.

10.7717/peerj.10809/supp-7 Supplemental Information 7 Environmental metadata and *nifH* qPCR and RT-qPCR derived abundances of UCYN-A1 and UCYN-A2 from each sampling site within Spencer Gulf and the adjacent continental shelf waters

## Additional Information and Declarations

### DNA Deposition

The following information was supplied regarding the deposition of DNA sequences: The sequences are available at the NCBI Sequence Read Archive: PRJNA294637, SAMN14858932-SAMN14858941. NifH sequences data are available at FigShare: - Messer, Lauren (2020): NifH qPCR/RTqPCR and environmental data from a temperate inverse estuary. figshare. Dataset. https://doi.org/10.6084/m9.figshare.12344312.v1.

### Data Availability

The following information was supplied regarding data availability: Raw data are available in the Supplemental Files and at FigShare: - Messer, Lauren (2020): NifH sequences from a temperate inverse estuary in southern Australia. figshare. Dataset. https://doi.org/10.6084/m9.figshare.12279743.v1
- Messer, Lauren (2020): NifH qPCR/RTqPCR and environmental data from a temperate inverse estuary. figshare. Dataset. https://doi.org/10.6084/m9.figshare.12344312.v1
- Messer, Lauren (2020): N2 fixation raw and rate data from Spencer Gulf South Australia. figshare. Dataset. https://doi.org/10.6084/m9.figshare.12961097.v1

## References

[ref-1] Ahmed A, Naik H, Adel SS, Bardhan P, Gauns M, Naik B, Naqvi S (2019). Nitrogen fixation and carbon uptake in a tropical estuarine system of Goa, western India. Journal of Sea Research.

[ref-2] Bentzon-Tilia M, Farnelid H, Jürgens K, Riemann L (2014). Cultivation and isolation of N2-fixing bacteria from suboxic waters in the Baltic Sea. FEMS Microbiology Ecology.

[ref-3] Bentzon-Tilia M, Severin I, Hansen LH (2015). Genomics and ecophysiology of heterotrophic nitrogen-fixing bacteria isolated from estuarine surface water. MBio.

[ref-4] Bentzon-Tilia M, Traving SJ, Mantikci M, Knudsen-Leerbeck H, Hansen JLS, Markager S, Riemann L (2015). Significant N_2_ fixation by heterotrophs, photoheterotrophs and heterocystous cyanobacteria in two temperate estuaries. ISME Journal.

[ref-5] Bernhard AE, Tucker J, Giblin AE, Stahl DA (2007). Functionally distinct communities of ammonia-oxidizing bacteria along an estuarine salinity gradient. Environmental Microbiology.

[ref-6] Berthelot H, Bonnet S, Camps M, Grosso O, Moutin T (2015). Assessment of the dinitrogen released as ammonium and dissolved organic nitrogen by unicellular and filamentous marine diazotrophic cyanobacteria grown in culture. Frontiers in Marine Science.

[ref-7] Bhavya PS, Kumar S, Gupta GVM, Sudheesh V, Sudharma KV, Varrier DS, Dhanya KR, Saravanane N (2016). Nitrogen uptake dynamics in a tropical eutrophic estuary (Cochin, India) and adjacent coastal waters. Estuaries and Coasts.

[ref-8] Bird C, Wyman M (2013). Transcriptionally active heterotrophic diazotrophs are widespread in the upper water column of the Arabian Sea. FEMS Microbiology Ecology.

[ref-9] Bombar D, Paerl RW, Riemann L (2016). Marine non-cyanobacterial diazotrophs: moving beyond molecular detection. Trends in Microbiology.

[ref-10] Bonnet S, Rodier M, Turk-Kubo KA, Germineaud C, Menkes C, Ganachaud A, Cravatte S, Raimbault P, Campbell E, Quéroué F, Sarthou G, Desnues A, Maes C, Eldin G (2015). Contrasted geographical distribution of N_2_ fixation rates and nifH phylotypes in the Coral and Solomon Seas (southwestern Pacific) during austral winter conditions. Global Biogeochem. Cycles.

[ref-11] Böttjer D, Dore JE, Karl DM, Letelier RM, Mahaffey C, Wilson ST, Zehr J, Church MJ (2017). Temporal variability of nitrogen fixation and particulate nitrogen export at Station ALOHA. Limnology and Oceanography.

[ref-12] Bouvier T, Del Giorgio P (2002). Compositional changes in free-living bacterial communities along a salinity gradient in two temperate estuaries. Limnology and Oceanography.

[ref-13] Breitbarth E, Oschlies A, LaRoche J (2007). Physiological constraints on the global distribution of Trichodesmium—effect of temperature on diazotrophy. Biogeosciences.

[ref-14] Brown MM, Friez MJ, Lovell CR (2003). Expression of *nifH* genes by diazotrophic bacteria in the rhizosphere of short form Spartina alterniflora. FEMS Microbiology Ecology.

[ref-15] Caffin M, Berthelot H, Cornet-Barthaux V, Barani A, Bonnet S (2018). Transfer of diazotroph-derived nitrogen to the planktonic food web across gradients of N_2_ fixation activity and diversity in the western tropical South Pacific Ocean. Biogeosciences.

[ref-16] Capone DG, Burns JA, Montoya JP, Subramaniam A, Mahaffey C, Gunderson T, Michaels AF, Carpenter EJ (2005). Nitrogen fixation by *Trichodesmium* spp.: an important source of new nitrogen to the tropical and subtropical North Atlantic Ocean. Global Biogeochemical Cycles.

[ref-17] Caporaso J, Bittinger K, Bushman FD, Desantis TZ, Andersen GL, Knight R (2010a). PyNAST: a flexible tool for aligning sequences to a template alignment. Bioinformatics.

[ref-18] Caporaso J, Kuczynski J, Stombaugh J, Bittinger K, Bushman F, Costello EK, Fierer N, Gonzalez Pena A, Goodrich J, Gordon JI, Huttley G, Kelley ST (2010b). QIIME allows analysis of high-throughput community sequencing data. Nature Methods.

[ref-19] Chen T-Y, Chen YL, Sheu D-S, Chen H-Y, Lin Y-H, Shiozaki T (2018). Community and abundance of heterotrophic diazotrophs in the northern South China Sea: revealing the potential importance of a new alphaproteobacterium in N_2_ fixation. Deep-Sea Research Part I: Oceanographic Research Papers.

[ref-20] Condie SA, Dunn JR (2006). Seasonal characteristics of the surface mixed layer in the Australasian region: implications for primary production regimes and biogeography. Marine and Freshwater Research.

[ref-21] Cornejo-Castillo FM, Muñoz Marín MDC, Turk-Kubo KA, Royo-Llonch M, Farnelid H, Acinas SG, Zehr J (2018). UCYN-A3, a newly characterized open ocean sublineage of the symbiotic N_2_-fixing cyanobacterium Candidatus Atelocyanobacterium thalassa. Environmental Microbiology.

[ref-22] Dabundo R, Lehmann MF, Treibergs L, Tobias CR, Altabet MA, Moisander PH, Granger J (2014). The Contamination of Commercial 15N2 Gas Stocks with 15N—Labeled Nitrate and Ammonium and Consequences for Nitrogen Fixation Measurements. PLOS ONE.

[ref-23] Delmont TO, Quince C, Shaiber A, Esen ÖC, Lee ST, Rappé MS, MacLellan SL, Lücker S, Eren AM (2018). Nitrogen-fixing populations of Planctomycetes and Proteobacteria are abundant in surface ocean metagenomes. Nature Microbiology.

[ref-24] Deloitte Access Economics (2017). The economic contribution of South Australia’s Marine Industries. Deloitte Touche Tohmatsu, November, 2017. http://www.misa.net.au/__data/assets/pdf_file/0004/324949/DAE_South_Australias_Marine_Industries_Print_May18.pdf.

[ref-25] Díez B, Bergman B, Pedrós-Alió C, Antó M, Snoeijs P (2012). High cyanobacterial *nifH* gene diversity in Arctic seawater and sea ice brine. Environmental Microbiology Reports.

[ref-26] Doubell MJ, Spencer D, Van Ruth PD, Lemckert C, Middleton JF (2018). Observations of vertical turbulent nitrate flux during summer in the Great Australian Bight. Deep-Sea Research Part II: Topical Studies in Oceanography.

[ref-27] Edgar RC (2010). Search and clustering orders of magnitude faster than BLAST. Bioinformatics.

[ref-28] Eugster O, Gruber N (2012). A probabilistic estimate of global marine N_2_-fixation and denitrification. Global Biogeochemical Cycles.

[ref-29] Eyre B (1998). Transport, retention and transformation of material in Australian Estuaries. Estuaries.

[ref-30] Farnelid H, Andersson AF, Bertilsson S, Al-Soud WA, Hansen LH, Sørensen S, Steward GF, Hagström Å, Riemann L (2011). Nitrogenase gene amplicons from global marine surface waters are dominated by genes of non-cyanobacteria. PLOS ONE.

[ref-31] Farnelid H, Bentzon-Tilia M, Andersson AF, Bertilsson S, Jost G, Labrenz M, Jürgens K, Riemann L (2013). Active nitrogen-fixing heterotrophic bacteria at and below the chemocline of the central Baltic Sea. ISME Journal.

[ref-32] Farnelid H, Harder J, Bentzon-Tilia M, Riemann L (2014). Isolation of heterotrophic diazotrophic bacteria from estuarine surface waters. Environmental Microbiology.

[ref-33] Farnelid H, Turk-Kubo K, Muñoz-Marín MC, Zehr JP (2016). New insights into the ecology of the globally significant uncultured nitrogen-fixing symbiont UCYN-A. Aquatic Microbial Ecology.

[ref-34] Fernandes M, Lauer P, Cheshire A, Angove M (2007). Preliminary model of nitrogen loads from southern bluefin tuna aquaculture. Marine Pollution Bulletin.

[ref-35] Fernandez C, Lorena González M, Munoz C, Molina V, Farías L (2015). Temporal and spatial variability of biological nitrogen fixation off the upwelling systemof central Chile (35–38.5oS). Journal of Geophysical Research: Oceans.

[ref-36] Fernández-Méndez M, Turk-Kubo KA, Buttigieg PL, Rapp JZ, Krumpen T, Zehr JP, Boetius A (2016). Diazotroph diversity in the sea ice, melt ponds, and surface waters of the eurasian basin of the Central Arctic Ocean. Frontiers in Microbiology.

[ref-37] Fish JA, Chai B, Wang Q, Sun Y, Brown CT, Tiedje JM, Cole JR (2013). FunGene: the functional gene pipeline and repository. Frontiers in Microbiology.

[ref-38] Fonseca-Batista D, Li X, Riou V, Michotey V, Deman F, Fripiat F, Guasco S, Brion N, Lemaitre N, Tonnard M, Gallinari M, Planquette H, Planchon F, Sarthou G, Elskens M, Laroche J, Chou L, Dehairs F (2019). Evidence of high N_2_ fixation rates in the temperate northeast Atlantic. Biogeosciences.

[ref-39] Foster RA, Kuypers MMM, Vagner T, Paerl RW, Musat N, Zehr J (2011). Nitrogen fixation and transfer in open ocean diatom-cyanobacterial symbioses. ISME Journal.

[ref-40] Foster RA, Subramaniam A, Mahaffey C, Carpenter EJ, Capone DG, Zehr J (2007). Influence of the Amazon River plume on distributions of free-living and symbiotic cyanobacteria in the western tropical north Atlantic Ocean. Limnology and Oceanography.

[ref-41] Gam ZBA, Oueslati R, Abdelkafi S, Casalot L, Tholozan JL, Labat M (2009). Desulfovibrio tunisiensis sp. nov. a novel weakly halotolerant, sulfate-reducing bacterium isolated from exhaust water of a Tunisian oil refinery. International Journal of Systematic and Evolutionary Microbiology.

[ref-42] Garcia N, Raimbault P, Sandroni V (2007). Seasonal nitrogen fixation and primary production in the Southwest Pacific: Nanoplankton diazotrophy and transfer of nitrogen to picoplankton organisms. Marine Ecology Progress Series.

[ref-43] Glibert PM, Bronk DA (1994). Release of dissolved organic nitrogen by marine diazotrophic cyanobacteria, Trichodesmium spp. Applied and Environmental Microbiology.

[ref-44] Goebel NL, Turk KA, Achilles KM, Paerl R, Hewson I, Morrison AE, Montoya JP, Edwards CA, Zehr J (2010). Abundance and distribution of major groups of diazotrophic cyanobacteria and their potential contribution to N_2_ fixation in the tropical Atlantic Ocean. Environmental Microbiology.

[ref-45] Gradoville MR, Bombar D, Crump BC, Letelier RM, Zehr JP, White AE (2017). Diversity and activity of nitrogen-fixing communities across ocean basins. Limnology and Oceanography.

[ref-46] Großkopf T, Mohr W, Baustian T, Schunck H, Gill D, Kuypers MMM, Lavik G, Schmitz RA, Wallace DWR, LaRoche J (2012). Doubling of marine dinitrogen-fixation rates based on direct measurements. Nature.

[ref-47] Harding K, Turk-Kubo KA, Sipler RE, Mills MM, Bronk DA, Zehr J (2018). Symbiotic unicellular cyanobacteria fix nitrogen in the Arctic Ocean. Proceedings of the National Academy of Sciences of the United States of America.

[ref-48] Heller P, Tripp HJ, Turk-Kubo K, Zehr J (2014). ARBitrator: a software pipeline for on-demand retrieval of auto-curated nifH sequences from GenBank. Bioinformatics.

[ref-49] Howarth RW, Marino R, Lane J, Cole JJ (1988). Nitrogen fixation in freshwater, estuarine, and marine ecosystems. 1. Rates and importance. Limnology and Oceanography.

[ref-50] Jayakumar A, Chang BX, Widner B, Bernhardt P, Mulholland MR, Ward BB (2017). Biological nitrogen fixation in the oxygen-minimum region of the eastern tropical North Pacific ocean. ISME Journal.

[ref-51] Jeffries TC, Schmitz Fontes ML, Harrison DP, Van-Dongen-Vogels V, Eyre BD, Ralph PJ, Seymour JR (2016). Bacterioplankton dynamics within a large anthropogenically impacted urban estuary. Frontiers in Microbiology.

[ref-52] Jickells TD (1998). Nutrient biogeochemistry of the coastal zone. Science.

[ref-53] Karl DM, Church MJ, Dore JE, Letelier RM, Mahaffey C (2012). Predictable and efficient carbon sequestration in the North Pacific Ocean supported by symbiotic nitrogen fixation. Proceedings of the National Academy of Sciences of the United States of America.

[ref-54] Karl D, Michaels A, Bergman B, Capone D, Carpenter E, Letelier R, Lipschultz F, Paerl H, Sigman D, Stal L (2002). Dinitrogen fixation in the world’s oceans. Biogeochemistry.

[ref-55] Kirchman DL, Dittel AI, Malmstrom RR, Cottrell MT (2005). Biogeography of major bacterial groups in the Delaware Estuary. Limnology and Oceanography.

[ref-56] Klawonn I, Lavik G, Böning P, Marchant HK, Dekaezemacker J, Mohr W, Ploug H (2015). Simple approach for the preparation of 15-15N2-enriched water for nitrogen fixation assessments: evaluation, application and recommendations. Frontiers in Microbiology.

[ref-57] Knapp AN (2012). The sensitivity of marine N2 fixation to dissolved inorganic nitrogen. Frontiers in Microbiology.

[ref-58] Krupke A, Mohr W, Laroche J, Fuchs BM, Amann RI, Kuypers MMM (2015). The effect of nutrients on carbon and nitrogen fixation by the UCYN-A—haptophyte symbiosis. ISME Journal.

[ref-59] Landolfi A, Koeve W, Dietze H, Kähler P, Oschlies A (2015). A new perspective on environmental controls of marine nitrogen fixation. Geophysical Research Letters.

[ref-60] Langlois R, Großkopf T, Mills M, Takeda S, LaRoche J (2015). Widespread distribution and expression of gamma A (UMB), an uncultured, diazotrophic, *γ*-proteobacterial *nifH* phylotype. PLOS ONE.

[ref-61] Langlois RJ, Hummer D, LaRoche J (2008). Abundances and distributions of the dominant *nifH* phylotypes in the Northern Atlantic Ocean. Applied and Environmental Microbiology.

[ref-62] Lauer PR, Fernandes M, Fairweather PG, Tanner J, Cheshire A (2009). Benthic fluxes of nitrogen and phosphorus at southern bluefin tuna *Thunnus maccoyii* sea-cages. The Marine Ecology Progress Series.

[ref-63] Lehnen N, Marchant HK, Schwedt A, Milucka J, Lott C, Weber M, Dekaezemacker J, Seah BKB, Hach PF, Mohr W, Kuypers MMM (2016). High rates of microbial dinitrogen fixation and sulfate reduction associated with the Mediterranean seagrass Posidonia oceanica. Systematic and Applied Microbiology.

[ref-64] Luo Y-W, Doney SC, Anderson LA, Benavides M, Berman-Frank I, Bode A, Bonnet S, Boström KH, Böttjer D, Capone DG, Carpenter EJ, Chen YL, Church MJ, Dore JE, Falcón LI, Fernández A, Foster RA, Furuya K, Gómez F, Gundersen K, Hynes AM, Karl DM, Kitajima S, Langlois RJ, LaRoche J, Letelier RM, Marañón E, McGillicuddy DJ, Moisander PH, Moore CM, Mouriño Carballido B, Mulholland MR, Needoba JA, Orcutt KM, Poulton AJ, Rahav E, Raimbault P, Rees AP, Riemann L, Shiozaki T, Subramaniam A, Tyrrell T, Turk-Kubo KA, Varela M, Villareal TA, Webb EA, White AE, Wu J, Zehr J (2012). Database of diazotrophs in global ocean: abundance, biomass and nitrogen fixation rates. Earth System Science Data.

[ref-65] Luo YW, Lima ID, Karl DM, Deutsch CA, Doney SC (2014). Data-based assessment of environmental controls on global marine nitrogen fixation. Biogeosciences.

[ref-66] Lynch TP, Morello EB, Evans K, Richardson AJ, Rochester W, Steinberg CR, Roughan M, Thompson P, Middleton JF, Feng M, Sherrington R, Brando V, Tilbrook B, Ridgway K, Allen S, Doherty P, Hill K, Moltmann TC (2014). IMOS National Reference Stations: a continental-wide physical, chemical and biological coastal observing system. PLOS ONE.

[ref-67] Martínez-Pérez C, Mohr W, Schwedt A, Dürschlag J, Callbeck CM, Schunck H, Dekaezemacker J, Buckner CRT, Lavik G, Fuchs BM, Kuypers MMM (2018). Metabolic versatility of a novel N2-fixing Alphaproteobacterium isolated from a marine oxygen minimum zone. Environmental Microbiology.

[ref-68] Messer LF, Brown MV, Furnas MJ, Carney RL, McKinnon AD, Seymour JR (2017). Diversity and activity of diazotrophs in great barrier reef surface waters. Frontiers in Microbiology.

[ref-69] Messer LF, Doubell M, Jeffries TC, Brown MV, Seymour JR (2015). Prokaryotic and diazotrophic population dynamics within a large oligotrophic inverse estuary. Aquatic Microbial Ecology.

[ref-70] Messer LF, Mahaffey C, Robinson CM, Jeffries TC, Baker KG, Isaksson JB, Ostrowski M, Doblin MA, Brown MV, Seymour JR (2016). High levels of heterogeneity in diazotroph diversity and activity within a putative hotspot for marine nitrogen fixation. ISME Journal.

[ref-71] Middleton JF, Bye JAT (2007). A review of the shelf-slope circulation along Australia’s southern shelves: cape Leeuwin to Portland. Progress in Oceanography.

[ref-72] Middleton J, Doubell M, James C, Luick J, Van Ruth P (2013). PIRSA Initiative II: carrying capacity of Spencer Gulf: hydrodynamic and biogeochemical measurement modelling and performance monitoring. Final Report for the Fisheries Research and Development Corporation.

[ref-73] Mills MM, Turk-Kubo KA, Van Dijken GL, Henke BA, Harding K, Wilson ST, Arrigo KR, Zehr J (2020). Unusual marine cyanobacteria/haptophyte symbiosis relies on N2 fixation even in N-rich environments. ISME Journal.

[ref-74] Mohr W, Großkopf T, Wallace DWR, LaRoche J (2010). Methodological underestimation of oceanic nitrogen fixation rates. PLOS ONE.

[ref-75] Moisander PH, Beinart RA, Hewson I, White AE, Johnson KS, Carlson CA, Montoya JP, Zehr JP (2010). Unicellular cyanobacterial distributions broaden the oceanic N_2_ fixation domain. Science.

[ref-76] Moisander PH, Serros T, Paerl RW, Beinart RA, Zehr J (2014). Gammaproteobacterial diazotrophs and nifH gene expression in surface waters of the South Pacific Ocean. ISME Journal.

[ref-77] Monteiro FM, Dutkiewicz S, Follows MJ (2011). Biogeographical controls on the marine nitrogen fixers. Global Biogeochemical Cycles.

[ref-78] Montoya J, Holl C, Zehr J, Hansen A (2004). High rates of N_2_ fixation by unicellular diazotrophs in the oligotrophic Pacific Ocean. Nature.

[ref-79] Montoya JP, Voss M, Kahler P, Capone DG (1996). A simple, high-precision, high-sensitivity tracer assay for N2 fixation. Applied and Environmental Microbiology.

[ref-80] Moore CM, Mills MM, Arrigo KR, Berman-Frank I, Bopp L, Boyd PW, Galbraith ED, Geider RJ, Guieu C, Jaccard SL, Jickells TD, LaRoche J, Lenton TM, Mahowald NM, Marañón E, Marinov I, Moore JK, Nakatsuka T, Oschlies A, Saito MA, Thingstad TF, Tsuda A, Ulloa O (2013). Processes and patterns of oceanic nutrient limitation. Nature Geoscience.

[ref-81] Mulholland MR (2007). The fate of nitrogen fixed by diazotrophs in the ocean. Biogeosciences.

[ref-82] Mulholland MR, Bernhardt PW, Blanco-Garcia JL, Mannino A, Hyde K, Mondragon E, Turk K, Moisander PH, Zehr J (2012). Rates of dinitrogen fixation and the abundance of diazotrophs in North American coastal waters between Cape Hatteras and Georges Bank. Limnology and Oceanography.

[ref-83] Mulholland MR, Bernhardt PW, Widner BN, Selden CR, Chappell PD, Clayton S, Mannino A, Hyde K (2019). High rates of N_2_ fixation in temperate, western North Atlantic coastal waters expands the realm of marine diazotrophy. Global Biogeochemical Cycles.

[ref-84] Mulholland MR, Bronk DA, Capone DG (2004). Dinitrogen fixation and release of ammonium and dissolved organic nitrogen by *Trichodesmium* IMS101. Aquatic Microbial Ecology.

[ref-85] Needoba JA, Foster RA, Sakamoto C, Zehr JP, Johnson KS (2007). Nitrogen fixation by unicellular diazotrophic cyanobacteria in the temperate oligotrophic North Pacific Ocean. Limnology and Oceanography.

[ref-86] Nunes RA, Lennon GW (1986). Physical property distributions and seasonal trends in Spencer Gulf, South Australia: an inverse estuary. Marine and Freshwater Research.

[ref-87] Nunes Vaz RA, Lennon GW, Bowers DG (1990). Physical behaviour of a large, negative or inverse estuary. Continental Shelf Research.

[ref-88] Paerl HW, Crocker KM, Prufert LE (1987). Limitation of N_2_ fixation in coastal marine waters: Relative importance of molybdenum, iron, phosphorus, and organic matter availability. Limnology and Oceanography.

[ref-89] Paxinos R (2007). Dynamics of phytoplankton in relation to tuna fish farms in Boston Bay and near-shore Spencer Gulf, South Australia. PhD Thesis.

[ref-90] Petrusevics PM (1993). SST fronts in inverse estuaries, South Australia- indicators of reduced gulf-shelf exchange. Australian Journal of Marine and Freshwater Research.

[ref-91] Petrusevics P, Bye J, Luick J, Teixeira CE (2011). Summer sea surface temperature fronts and elevated chlorophyll-a in the entrance to Spencer Gulf, South Australia. Continental Shelf Research.

[ref-92] Pritchard D (1952). Estuarine hydrography. Advances in Geophysics.

[ref-93] R Core Team (2013). http://www.R-project.org/.

[ref-94] Raes EJ, Thompson PA, McInnes AS, Nguyen HM, Hardman-Mountford N, Waite AM (2015). Sources of new nitrogen in the Indian Ocean. Global Biogeochemical Cycles.

[ref-95] Raes EJ, Waite AM, McInnes AS, Olsen H, Nguyen HM, Hardman-Mountford N, Thompson PA (2014). Changes in latitude and dominant diazotrophic community alter N_2_ fixation. Marine Ecology Progress Series.

[ref-96] Rees AP, Gilbert JA, Kelly-Gerreyn BA (2009). Nitrogen fixation in the western English Channel (NE Atlantic Ocean). Marine Ecology Progress Series.

[ref-97] Rees AP, Law CS, Woodward EMS (2006). High rates of nitrogen fixation during an in-situ phosphate release experiment in the Eastern Mediterranean Sea. Geophysical Research Letters.

[ref-98] Rivero-Calle S, Del Castillo CE, Gnanadesikan A, Dezfuli A, Zaitchik B, Johns DG (2016). Interdecadal Trichodesmium variability in cold North Atlantic waters. Global Biogeochemical Cycles.

[ref-99] Robbins WD, Huveneers C, Parra GJ, Möller L, Gillanders BM (2017). Anthropogenic threat assessment of marine-associated fauna in Spencer Gulf, South Australia. Marine Policy.

[ref-100] Sañudo Wilhelmy SA, Kustka AB, Gobler CJ, Hutchins DA, Yang M, Lwiza K, Burns J, Capone DG, Raven JA, Carpenter EJ (2001). Phosphorus limitation of nitrogen fixation by *Trichodesmium* in the central Atlantic Ocean. Nature.

[ref-101] Schlitzer R (2018). https://odv.awi.de.

[ref-102] Shiozaki T, Bombar D, Riemann L, Hashihama F, Takeda S, Yamaguchi T, Ehama M, Hamasaki K, Furuya K (2017). Basin scale variability of active diazotrophs and nitrogen fixation in the North Pacific, from the tropics to the subarctic Bering Sea. Global Biogeochem. Cycles.

[ref-103] Shiozaki T, Nagata T, Ijichi M, Furuya K (2015a). Nitrogen fixation and the diazotroph community in the temperate coastal region of the northwestern North Pacific. Biogeosciences.

[ref-104] Shiozaki T, Nagata T, Ijichi M, Furuya K (2015b). Seasonal dynamics of nitrogen fixation and the diazotroph community in the temperate coastal region of the northwestern North Pacific. Biogeosciences Discuss.

[ref-105] Short SM, Zehr J (2007). Nitrogenase gene expression in the Chesapeake Bay Estuary. Environmental Microbiology.

[ref-106] Singh A, Gandhi N, Ramesh R (2019). Surplus supply of bioavailable nitrogen through N_2_ fixation to primary producers in the eastern Arabian Sea during autumn. Continental Shelf Research.

[ref-107] Smith SV, Veeh HH (1989). Mass balance of biogeochemically active materials (C, N, P) in a hypersaline gulf. Estuar. Coast. Estuarine, Coastal and Shelf Science.

[ref-108] Stenegren M, Caputo A, Berg C, Bonnet S, Foster RA (2018). Distribution and drivers of symbiotic and free-living diazotrophic cyanobacteria in the western tropical South Pacific. Biogeosciences.

[ref-109] Tang W, Li Z, Cassar N (2019). Machine learning estimates of global marine nitrogen fixation. Journal of Geophysical Research: Biogeosciences.

[ref-110] Tang W, Wang S, Fonseca-Batista D, Dehairs F, Gifford S, Gonzalez AG, Gallinari M, Planquette H, Sarthou G, Cassar N (2019). Revisiting the distribution of oceanic N_2_ fixation and estimating diazotrophic contribution to marine production. Nature Communications.

[ref-111] Taniuchi Y, Chen YLL, Chen HY, Tsai ML, Ohki K (2012). Isolation and characterization of the unicellular diazotrophic cyanobacterium Group C TW3 from the tropical western Pacific Ocean. Environmental Microbiology.

[ref-112] Thompson A, Carter BJ, Turk-Kubo K, Malfatti F, Azam F, Zehr J (2014). Genetic diversity of the unicellular nitrogen-fixing cyanobacteria UCYN-A and its prymnesiophyte host. Environmental Microbiology.

[ref-113] Turk-Kubo KA, Achilles KM, Serros TRC, Ochiai M, Montoya JP, Zehr J (2012). Nitrogenase (*nifH*) gene expression in diazotrophic cyanobacteria in the Tropical North Atlantic in response to nutrient amendments. Frontiers in Microbiology.

[ref-114] Turk-Kubo KA, Farnelid HM, Shilova IN, Henke B, Zehr J (2016). Distinct ecological niches of marine symbiotic N_2_-fixing cyanobacterium Candidatus atelocyanobacterium thalassa sublineages. Journal of Phycology.

[ref-115] Turk-Kubo KA, Karamchandani M, Capone DG, Zehr J (2014). The paradox of marine heterotrophic nitrogen fixation: abundances of heterotrophic diazotrophs do not account for nitrogen fixation rates in the Eastern Tropical South Pacific. Environmental Microbiology.

[ref-116] Van Ruth PD, Patten NL, Doubell MJ, Chapman P, Rodriguez AR, Middleton JF (2018). Seasonal- and event-scale variations in upwelling, enrichment and primary productivity in the eastern Great Australian Bight. Deep-Sea Research Part II: Topical Studies in Oceanography.

[ref-117] Wang WL, Moore JK, Martiny AC, Primeau FW (2019). Convergent estimates of marine nitrogen fixation. Nature.

[ref-118] Wang Y, Naumann U, Wright ST, Warton DI (2012). Mvabund—an R package for model-based analysis of multivariate abundance data. Methods in Ecology and Evolution.

[ref-119] Wannicke N, Benavides M, Dalsgaard T, Dippner JW, Montoya JP, Voss M (2018). New perspectives on nitrogen fixation measurements using 15N2 gas. Frontiers in Marine Science.

[ref-120] Ward BA, Dutkiewicz S, Moore CM, Follows MJ (2013). Iron, phosphorus, and nitrogen supply ratios define the biogeography of nitrogen fixation. Limnology and Oceanography.

[ref-121] Warthmann R, Vasconcelos C, Sass H, McKenzie JA (2005). Desulfovibrio brasiliensis sp. nov. a moderate halophilic sulfate-reducing bacterium from Lagoa Vermelha (Brazil) mediating dolomite formation. Extremophiles.

[ref-122] Watkins-Brandt KS, Letelier RM, Spitz YH, Church MJ, Böttjer D, White AE (2011). Addition of inorganic or organic phosphorus enhances nitrogen and carbon fixation in the oligotrophic North Pacific. Marine Ecology Progress Series.

[ref-123] Weber SC, Carpenter EJ, Coles VJ, Yager PL, Goes J, Montoya J (2017). Amazon River influence on nitrogen fixation and export production in the western tropical North Atlantic. Limnology and Oceanography.

[ref-124] White AE, Granger J, Selden C, Gradoville MR, Potts L, Bourbonnais A, Fulweiler RW, Knapp AN, Mohr W, Moisander PH, Tobias CR, Caffin M, Wilson ST, Benavides M, Bonnet S, Mulholland MR, Chang BX (2020). A critical review of the 15N2 tracer method to measure diazotrophic production in pelagic ecosystems. Limnology and Oceanography: Methods.

[ref-125] Wilson ST, Bottjer D, Church MJ, Karl DM (2012). Comparative assessment of nitrogen fixation methodologies, conducted in the oligotrophic North Pacific Ocean. Applied and Environmental Microbiology.

[ref-126] Zani S, Mellon MT, Collier JL, Zehr J (2000). Expression of *nifH* genes in natural microbial assemblages in Lake George, New York, detected by reverse transcriptase PCR. Applied and Environmental Microbiology.

[ref-127] Zehr JP, Carpenter EJ, Villareal TA (2000). New perspectives on nitrogen-fixing microorganisms in tropical and subtropical oceans. Trends in Microbiology.

[ref-128] Zehr JP, Jenkins BD, Short SM, Steward GF (2003). Nitrogenase gene diversity and microbial community structure: a cross system comparison. Environmental Microbiology.

[ref-129] Zehr JP, Kudela RM (2011). Nitrogen cycle of the open ocean: from genes to ecosystems. Annual Review of Marine Science.

[ref-130] Zehr JP, Mellon MT, Zani S, York N (1998). New Nitrogen-Fixing Microorganisms Detected in Oligotrophic Oceans by Amplification of Nitrogenase (*nifH*) Genes. Applied and Environmental Microbiology.

[ref-131] Zehr JP, Turner PJ (2001). Nitrogen fixation: nitrogenase genes and gene expression. Methods of Microbiology and Molecular Biology.

