# Supplementary material for: Temperate southern Australian coastal waters are characterised by surprisingly high rates of nitrogen fixation and diversity of diazotrophs
Source: PeerJ. 2021 Mar 1;9:e10809. doi: 10.7717/peerj.10809 (PMC7931716; doi:10.7717/peerj.10809)
Supplement: Table S1 [file peerj-09-10809-s001.docx]

| *nifH* target | Forward primer (5'-3') | Probe (5'-3') | Reverse primer (5'-3') |
| --- | --- | --- | --- |
| UCYN-A1^1^ | TAGCTGCAGAAAGAGGAACTGTAGAAG | TAATTCCTGGCTATAACAAC | TCAGGACCACCGGACTCAAC |
| UCYN-A2^2^ | GGTTACAACAACGTTTTATGTGTTGA | TCTGGTGGTCCTGAGCCCGGA | ACCACGACCAGCACATCCA |

1 Langlois, R.J., Hümmer, D., LaRoche, J., 2008. Abundances and distributions of the dominant *nifH* phylotypes in the Northern Atlantic Ocean. *Appl. Environ. Microbiol.,* 74(6):1922–31.

2 Thompson, A. et al., 2014. Genetic diversity of the unicellular nitrogen-fixing cyanobacteria UCYN-A and its prymnesiophyte host. *Environ. Microbiol.*, 16(10):3238–3249
