# Supplementary material for: Temperate southern Australian coastal waters are characterised by surprisingly high rates of nitrogen fixation and diversity of diazotrophs
Source: PeerJ. 2021 Mar 1;9:e10809. doi: 10.7717/peerj.10809 (PMC7931716; doi:10.7717/peerj.10809)
Supplement: Table S2 [file peerj-09-10809-s002.docx]

| **Site** | **H’ Autumn** | **H’ Summer** |
| --- | --- | --- |
| Shelf | 1.95 | 2.86 |
| Mouth | 2.99 | 2.89 |
| S-Gulf | 4.11 | 3.38 |
| M-Gulf | 4.97 | 4.37 |
| N-Gulf | 4.31 | 4.40 |
| Mean | 3.67 | 3.58 |
