# Supplementary material for: Temperate southern Australian coastal waters are characterised by surprisingly high rates of nitrogen fixation and diversity of diazotrophs
Source: PeerJ. 2021 Mar 1;9:e10809. doi: 10.7717/peerj.10809 (PMC7931716; doi:10.7717/peerj.10809)
Supplement: Table S3 — nifH subclusters, and the closest cultured representatives, were determined by the FunGene pipeline. AAI = amino acid identity. [file peerj-09-10809-s003.docx]

| OTU ID | Closest Representative (*nifH* RDP) | Amino Acid Identity % |
| --- | --- | --- |
| OTU7980 | *Candidatus* Atelocyanobacterium thalassa UCYN-A1 | 91 |
| OTU3535 | *Candidatus* Atelocyanobacterium thalassa UCYN-A1 | 97 |
| OTU1115 | *Candidatus* Atelocyanobacterium thalassa UCYN-A1 | 91 |
| OTU45147 | *Candidatus* Atelocyanobacterium thalassa UCYN-A1 | 92 |
| OTU51120 | *Candidatus* Atelocyanobacterium thalassa UCYN-A1 | 99 |
| OTU9097 | *Candidatus* Atelocyanobacterium thalassa UCYN-A2 | 100 |
| OTU67260 | *Candidatus* Atelocyanobacterium thalassa UCYN-A4 | 100 |
| OTU119322 | *Coraliomargarita akajimensis* DSM45221 | 85 |
| OTU46506 | *Desulfatibacillum alkenivorans* AK-01 | 87 |
| OTU78456 | *Desulfonatronospira thiodismutans* | 87 |
| OTU39174 | *Desulfovibrio aespoeensis* | 89 |
| OTU11657 | *Desulfovibrio aespoeensis* | 94 |
| OTU80903 | *Desulfovibrio aespoeensis* | 90 |
| OTU41624 | *Desulfovibrio aespoeensis* | 96 |
| OTU29599 | *Desulfovibrio magneticus* | 85 |
| OTU16465 | *Oscillatoria sp* PCC6506 | 92 |
| OTU105431 | *Pelobacter carbinolicus* DSM2380 | 90 |
| OTU83321 | *Pseudomonas stutzeri* | 92 |
| OTU35906 | *Pseudomonas stutzeri* | 96 |
| OTU33917 | *Synechococcus sp* PCC7335 | 97 |
| OTU13654 | *Thiorhodococcus drewsii* | 87 |
| OTU56913 | *Trichodesmium erythraeum* IMS101 | 98 |
| OTU121192 | *Verrucomicrobiae* | 83 |
| OTU85365 | *Verrucomicrobiae* | 84 |
| OTU59707 | *Verrucomicrobiae* | 85 |
