# Supplementary figures and images for: Temperate southern Australian coastal waters are characterised by surprisingly high rates of nitrogen fixation and diversity of diazotrophs
Source: PeerJ. 2021 Mar 1;9:e10809. doi: 10.7717/peerj.10809 (PMC7931716; doi:10.7717/peerj.10809)

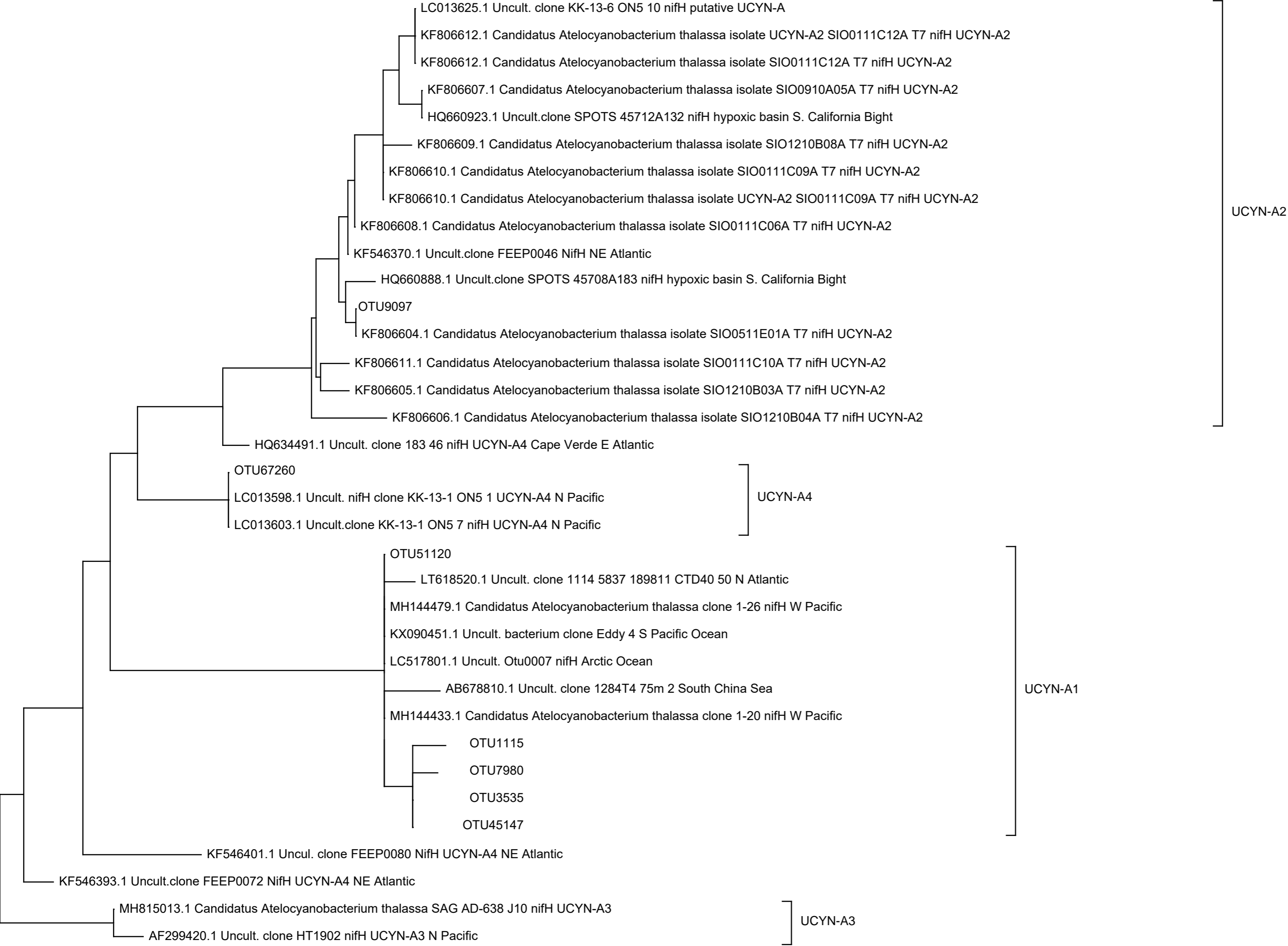

0.010

Supplement: Figure S1 — Tree inference was performed using the Maximum Likelihood method and Tamura-Nai model in MEGAX (v10.1.8; Kumar et al., 2018. The scale bar represents the number of substitutions per site. Kumar S., Stecher G., Li M., Knyaz C., and Tamura K. (2018). MEGA X: Molecular Evolutionary Genetics Analysis across computing platforms. Molecular Biology and Evolution 35:1547-1549. [file peerj-09-10809-s005.pdf]
